# Multi-omic profiling of lung and liver tumor microenvironments of metastatic pancreatic cancer reveals site-specific immune regulatory pathways

**DOI:** 10.1186/s13059-021-02363-6

**Published:** 2021-05-13

**Authors:** Won Jin Ho, Rossin Erbe, Ludmila Danilova, Zaw Phyo, Emma Bigelow, Genevieve Stein-O’Brien, Dwayne L. Thomas, Soren Charmsaz, Nicole Gross, Skylar Woolman, Kayla Cruz, Rebecca M. Munday, Neeha Zaidi, Todd D. Armstrong, Marcelo B. Sztein, Mark Yarchoan, Elizabeth D. Thompson, Elizabeth M. Jaffee, Elana J. Fertig

**Affiliations:** 1 Department of Oncology, Sidney Kimmel Comprehensive Cancer Center, Johns Hopkins School of Medicine, 550 N Broadway Suite 1101E, Baltimore, MD 21209 USA; 2 The Johns Hopkins Cancer Convergence Institute, Baltimore, USA; 3 Skip Viragh Center for Pancreatic Cancer, Bloomberg–Kimmel Institute for Cancer Immunotherapy, Johns Hopkins University School of Medicine, 4M07 Bunting Blaustein Cancer Research Building, 1650 Orleans Street, Baltimore, MD 21287 USA; 4 McKusick-Nathans Institute of Genetic Medicine, Johns Hopkins University, Baltimore, USA; 5 Department of Pathology, Johns Hopkins University School of Medicine, Baltimore, USA; 6 Center for Vaccine Development and Global Health, University of Maryland School of Medicine, Baltimore, MD USA; 7 Department of Applied Mathematics and Statistics, Johns Hopkins University Whiting School of Engineering, Baltimore, MD USA; 8 Department of Biomedical Engineering, Johns Hopkins University School of Medicine, Baltimore, USA

## Abstract

**Background:**

The majority of pancreatic ductal adenocarcinomas (PDAC) are diagnosed at the metastatic stage, and standard therapies have limited activity with a dismal 5-year survival rate of only 8%. The liver and lung are the most common sites of PDAC metastasis, and each have been differentially associated with prognoses and responses to systemic therapies. A deeper understanding of the molecular and cellular landscape within the tumor microenvironment (TME) metastasis at these different sites is critical to informing future therapeutic strategies against metastatic PDAC.

**Results:**

By leveraging combined mass cytometry, immunohistochemistry, and RNA sequencing, we identify key regulatory pathways that distinguish the liver and lung TMEs in a preclinical mouse model of metastatic PDAC. We demonstrate that the lung TME generally exhibits higher levels of immune infiltration, immune activation, and pro-immune signaling pathways, whereas multiple immune-suppressive pathways are emphasized in the liver TME. We then perform further validation of these preclinical findings in paired human lung and liver metastatic samples using immunohistochemistry from PDAC rapid autopsy specimens. Finally, in silico validation with transfer learning between our mouse model and TCGA datasets further demonstrates that many of the site-associated features are detectable even in the context of different primary tumors.

**Conclusions:**

Determining the distinctive immune-suppressive features in multiple liver and lung TME datasets provides further insight into the tissue specificity of molecular and cellular pathways, suggesting a potential mechanism underlying the discordant clinical responses that are often observed in metastatic diseases.

**Supplementary Information:**

The online version contains supplementary material available at 10.1186/s13059-021-02363-6.

## Introduction

Pancreatic cancer, mostly comprised of pancreatic ductal adenocarcinoma (PDAC), is now the third leading cause of death in the USA with over 45,000 deaths this year [1]. Over 70% of PDAC patients are diagnosed with metastatic disease at diagnosis, and the 5-year survival rate for these patients has only modestly risen to 10% [2]. PDAC most commonly metastasizes to liver and lung [3], and the site of metastatic involvement has prognostic implications. Specifically, multiple institutional reviews have observed that isolated pulmonary metastatic disease has significantly better prognosis than patients with liver metastases [4–6]. This clinical distinction is not unique to PDACs. Similar prognostic contrast has been noted for melanomas and lung cancers, and patients with liver metastases have poorer survival outcomes when treated with PD-1 immune checkpoint therapy when compared with patients who have disease involvement in other visceral sites including lung [7, 8].

These differences have generally been attributed to clonal heterogeneity of the tumor itself, as well as tissue-specific features of the tumor microenvironment (TME) [9]. A deeper understanding of the distinctive features within different metastatic TMEs has the potential to inform future therapeutic strategies and/or identify novel targets for therapy against metastatic PDAC. However, the ability to study these site-specific differences has been challenging for two reasons. First, patient samples from matched metastatic sites are generally of limited availability since there is rarely ever a need for simultaneous multisite biopsy for clinical decision-making, and second, animal models of sporadic metastases pose the logistic challenge of establishing controlled cohorts and are subject to clonal heterogeneity.

In this study, we employed intraportal/hemispleen and intravenous injection mouse models of hepatic and pulmonary metastatic PDAC, respectively, to directly compare the liver and lung TMEs of metastatic PDAC. We analyzed the immune compartment of the TMEs by high-dimensional mass cytometry and immunohistochemistry, and the non-immune, magnetically enriched compartment of the TMEs by bulk RNAseq. To correlate these observations with human disease, we also used five pairs of matched human liver and lung metastatic samples from our pancreatic cancer rapid autopsy biobank to perform immunohistochemistry evaluating canonical immune cell markers. Finally, we utilized our transfer learning methodology ProjectR [10] to computationally test whether the differences are site-driven by comparing lung and liver cancer in TCGA and additional metastatic models in MetMap [11]. Based on these investigations, we identified immune regulatory processes resulting from microenvironment and tumor cell interactions that are enriched in specific metastatic sites both at the tumor-bearing and normal baseline states, highlighting the importance of understanding the tissue-intrinsic factors that may impact tumor behavior.

## Results

### Establishing models of hepatic and pulmonary metastatic pancreatic ductal adenocarcinoma

To establish directly comparable lung and liver PDAC metastasis, we utilized a murine pancreatic adenocarcinoma cell line driven by *Kras* and *Trp53* mutations (KPC) [12] injected into the two most common visceral sites of metastatic disease, liver, and lung. The use of this cell line was particularly important given its genetic similarity to human disease [13] and reflects the critical impact that mutations have on the immune response [14]. Similar to human disease, KPC cells are thought to be intrinsically limited in immunogenicity. A relatively low mutational burden in our KPC cell line was verified by whole exome sequencing and mutational analysis (Additional file 1: Figure S1A). The carcinogen-induced mouse pancreatic cancer cell line Panc02, which is known to harbor a high number of mutations including those that yield immunologically recognizable neoantigens [15], was used as a comparator. As a gross assessment of neoantigens related to the driver mutations in the KPC model, predicted MHC binding affinities for *Kras*(*G12D*) and *Trp53*(*R172H*) were also analyzed; no mutations in either of these genes were identified in the Panc02 cell line. As expected, neither driver mutation produced a putatively strong neoantigen as defined by a strong binding affinity of the mutant type peptide or by comparison of the mutant type to wild type binding affinity (Additional file 1: Figure S1B, C). To establish metastasis, KPC cells were injected intraportally by the hemispleen method [16] and intravenously via tail vein, leading to robust tumor burdens in the liver and lung by day 21 from the time of injection that could be validated by both gross and histological examination (Additional file 1: Figure S2). Using these models, both the immune and non-immune compartments of lung and liver microenvironments were analyzed with high-throughput molecular profiling to fully interrogate the differences between the two metastatic tissue sites.

### Immune profiling with mass cytometry reveals enhanced immune activation in lung metastases

**Fig. 1:**
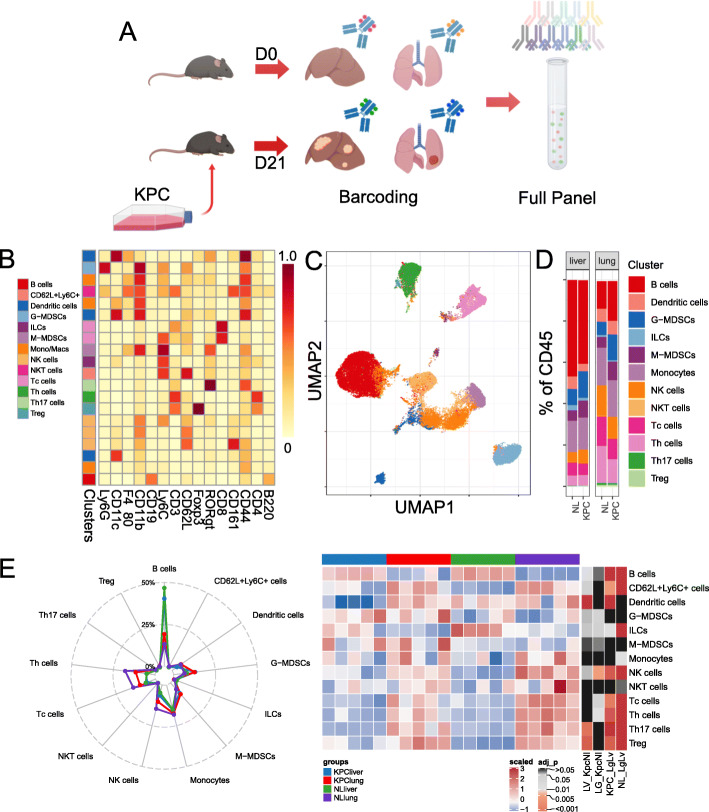
Global immune profiling of metastatic liver and lung TME with CyTOF. **a** Mice were injected with *Kras*G12D and *TP53*R172H-driven pancreatic cancer cells (KPC) into the liver by the hemispleen method or into the lung by intravenous tail vein injection. Normal lung and livers (day 0, “D0”) and day 21 (“D21”) from the day of injection were harvested and barcoded with a CD45 antibody conjugated to a unique metal. One sample from each group was then combined into a 4-plex batch to be stained with the full CyTOF panel (Table S1). **b** Heatmap of normalized marker expression for FlowSOM clustering of the dataset and **c** UMAP visualization of the clusters are shown. Two thousand events per sample are represented. Immune cell type proportions as a percentage of total CD45+ cells from each of the four groups are shown as **d** stacked barplots for the entire group and **e** mean values for each group as radar plots (left) and relative values scaled for each immune cell subtype for all mice as a heatmap (right). Groups are annotated by color within the radar plot or as horizontal bars above the heatmap. FDR-adjusted *p* values compared using *edgeR* for KPC-bearing vs. normal conditions in the liver (LV_KpcNl) and lung (LG_KpcNl) as well as lung vs. liver in KPC-bearing (KPC_LgLv) and normal (NL_LgLv) conditions are annotated as an adjacent heatmap to the right

To characterize the microenvironment of the metastases, we profiled the immune compartment of normal liver, normal lung, KPC-bearing liver, and KPC-bearing lung with mass cytometry (Cytometry by Time-of-Flight; CyTOF). The CyTOF panel consisted of a total of 33 mass channels, including 16 for subtyping, 11 for functional analysis, 4 for barcoding, 1 for cell identification, and 1 for viability (Additional file 2: Table S1). By using four unique CD45 barcodes, we employed a batching strategy to enable multiplexed staining and data acquisition for robust analysis (Fig. 1a). The high-dimensional CyTOF data was then clustered using FlowSOM algorithm into a total of 20 metaclusters, which were then annotated into 13 final immune cell types (Fig. 1b and Additional file 3: Table S2). Annotations were based on known canonical expression profile of the different immune cell types, e.g. CD3^+^CD4^+^ clusters are annotated as helper T cells. When comparing between the two metastatic sites, a higher proportion of B cells were found in liver while a higher proportion of T cells, NK cells, monocytes, and CD11c^+^ dendritic cells were found in lung. Most of these organ-specific differences were also present in the absence of KPC metastasis, reflecting differences in the baseline immune infiltrate of these tissues. As expected, higher proportions of myeloid derived suppressor cells (MDSCs) and Tregs were noted in the KPC-bearing states for both liver and lung (Fig. 1c–e). When we assessed the functional profiles within each of the cell type clusters, lung generally exhibited higher mean metal intensities (MMI) of activation or co-stimulatory markers, CD69, ICOS, and CD27, and co-inhibitory markers, PD-L1, PD1, KLRG1, and BTLA (Additional file 1: Figure S3). A notable exception to this trend was higher MMI of LAG3 in T cell and NK cell clusters within the liver.

**Fig. 2:**
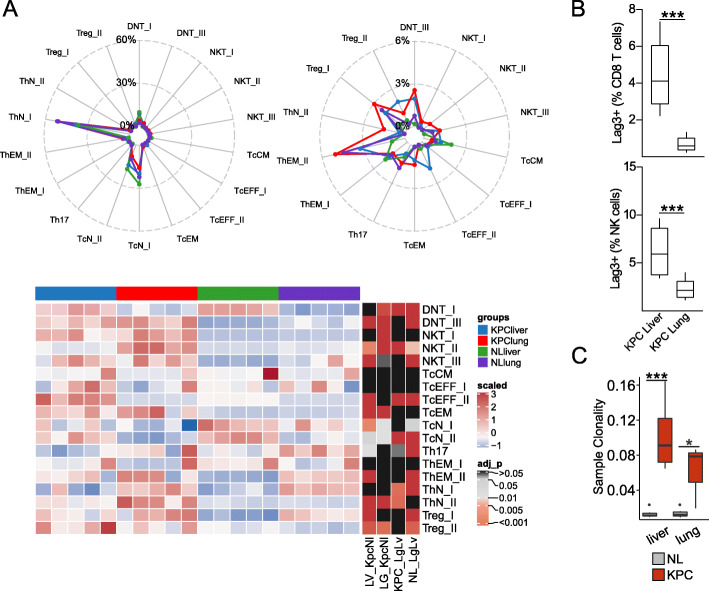
T cell profiling of metastatic liver and lung TME with CyTOF. **a** CD3+ subset of the dataset was re-clustered using both subtyping and functional markers. Select T cell clusters are represented with mean values for each group as radar plots (top). Separate radar plots with different axis ranges are used to facilitate the visualization of low-level subtypes. Relative values scaled for each immune cell subtype for all mice as a heatmap (bottom). Groups are annotated by color within the radar plot or as horizontal bars above the heatmap. FDR-adjusted *p* values compared using *edgeR* for KPC-bearing vs. normal conditions in the liver (LV_KpcNl) and lung (LG_KpcNl) as well as lung vs. liver in KPC-bearing (KPC_LgLv) and normal (NL_LgLv) conditions are annotated as an adjacent heatmap to the right. **b** Proportion of LAG3 positive CD8 T cells (top) and NK cells (bottom) as assessed by fluorescent flow cytometry comparing between KPC-bearing liver and lung is shown (*n* = 6). Two-tailed *t* test, ***p < 0.005. **c** Sample clonality values derived from TCRseq analysis of T cells within normal (gray) livers and lungs and KPC-bearing (red) livers and lungs are compared. Wilcoxon-Mann-Whitney, *p < 0.05; ***< 0.005

Given the increased numbers of T cells in the lung metastatic microenvironment, we repeated the clustering analysis utilizing samples gated for CD3^+^ events for deeper profiling of T cells. All functional markers were incorporated into the clustering step, and a total of 20 resulting metaclusters were annotated into 18 T cell subtypes based on their expression profiles (Additional file 1: Figure S4 and Additional file 4: Table S3). As a percent of CD3^+^ cells, a much higher proportion of naïve helper T cells were seen in the lung compared to the liver (Fig. 2a), driving most of the difference seen in the T cell percentage of all CD45^+^ immune cells (Additional file 1: Figure S5). Compared to the normal tissue controls, KPC-bearing tissues had greater proportions of regulatory T cell clusters (“Treg_I” and “Treg_II”) and PD-1-expressing effector memory T cell clusters (“TcEM” and “ThEM_II”) along with decreased proportion of naïve cytotoxic T cell clusters (“TcN_I” and “TcN_II”) (Fig. 2a). Another important distinction was the tumor-associated presence (2% of CD3^+^ T cells) of Lag3^+^ Granzyme B^+^ cytotoxic T cells (“TcEFF_II”) within the liver but not in the lung microenvironment (Fig. 2a). Since metal intensity of Lag3 was relatively weak and since the Lag3-positive population was rare, we then performed fluorescent flow cytometry to validate these findings. Again, we confirmed the higher percentage of Lag3^+^ CD8 T cells and NK cells in the KPC-bearing liver compared to the KPC-bearing lung (Fig. 2b). In addition, to assess whether these changes in the proportions of T cell subtypes are truly tumor-responsive, we performed TCR repertoire analysis of the tissue-infiltrating T cells in both normal and KPC-bearing livers and lungs. Compared to normal tissue controls, KPC-bearing livers and lungs demonstrated a significantly higher sample clonality of T cells (Fig. 2c), suggesting that the differences in the T cell subpopulations are actually driven by the presence of the tumors within these metastatic sites. Together, these findings implied that compared with the liver, the lung metastatic microenvironment is generally composed of a higher level of tumor-associated immune cell infiltration and activation, especially T cells.

To further validate that the differences in the immune cell profiles are not just tissue-intrinsic differences but also altered in ways that are specific to KPC tumors, we performed immunohistochemistry (IHC) of four immune cell markers, CD8 (cytotoxic T cells), CD4 (helper T cells), CD68 (macrophage/myeloid cells), and B220 (B cells), to visually assess their infiltration within the two tissue sites (Additional file 1: Figure S6A-F). We specified our analysis to tumoral and adjacent normal regions separately and first quantified the density of cells. When comparing the number of cells per tissue area — in contrast to % of cells in CyTOF — there were higher density of CD4^+^ T, CD68^+^ myeloid, and B220^+^ B cells within the lung tumors compared to the liver tumors (Additional file 1: Figure S6G). There was a trend toward higher density of CD8^+^ T cells as well. To further characterize their relationships, e.g. the proximity of an antitumor effector CD8^+^ T cell to all other immune cells, we also measured the average distances between any given CD8^+^ T cell and CD4^+^ T cells, CD68^+^ myeloid cells, B220^+^ B cells, or another CD8^+^ T cell (Additional file 1: Figure S6H). The shortest distances among immune cells were observed within the tumors in the lungs, suggesting that the spatial coordination of immune cells is different between the lung and the liver TMEs. Importantly, the immune cell densities and distances were also higher and shorter, respectively, in the tumoral regions than their normal adjacent counterparts, suggesting that the immunologic profiles are not only different between the two sites but also shaped differentially by the tumor.

### Transcriptomic analysis of the non-immune compartment implicate a role for liver parenchyma in establishing an immune-suppressive TME

**Fig. 3:**
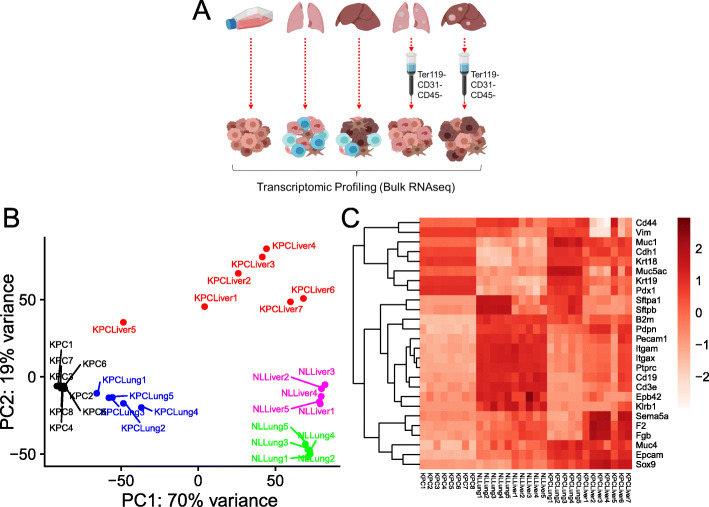
Approach for non-immune compartment profiling of metastatic liver and lung TME by RNAseq analysis. **a** KPC-bearing lung and liver are analyzed along with KPC cells from in vitro culture in 2D, normal lung, and normal liver as controls. All tissue samples are enzymatically dissociated into single cells. KPC-bearing liver and lung samples are further processed using negative magnetic bead selection to obtain the non-immune cells. **b** PCA plotting of the RNAseq dataset shows how individual samples cluster. **c** Expression of key genes identifying cells of pancreatic origin (KPC), tissue-specific parenchyma, and immune cells are shown in a heatmap to demonstrate the quality of the sample preparation process

To compare the features within the non-immune compartment, which includes the KPC cells, tissue-specific parenchymal cells, and stromal cells, KPC-bearing liver and lung samples were enzymatically dissociated into single cells and were subsequently processed by negative selection with a cocktail of magnetic beads targeting CD45 (pan-immune cells), CD31 (endothelial cells), and Ter119 (red blood cells) among others. The resulting samples were then analyzed by RNAseq along with KPC cells from 2D in vitro culture, normal lung samples, and normal liver samples as controls (Fig. 3a). PC analysis showed that samples from each of the groups clustered together (Fig. 3b). PC1 represented 71% of the variance and associated mostly with the contrast between KPC-bearing and normal samples, whereas PC2 represented 19% of the variance and associated with tissue type. Comparing the expression of KPC-associated genes, *Pdx1*, *Muc1*, *Muc5ac*, *Sox9*, *Krt18*, *Krt19*, and *Cdh1* confirmed that the method successfully selected for KPC cells (Fig. 3c). Furthermore, the presence of parenchymal cell markers for liver (prothrombin and fibrinogen genes; *F2*, *Fgb*) and lung (surfactant protein genes; *Sftpa1*, *Sftpb*) was represented by the corresponding liver and lung samples, respectively, and not by the in vitro KPC cells. Also, substantial reduction of immune cells (*Ptprc*, *CD3e*, *CD19* expression) in the KPC-bearing livers and lungs by the negative selection process was confirmed (Fig. 3c).

Next, to assess the signaling pathways within the non-immune compartment that may be interacting with the immune compartment, genes significantly different between the liver and lung non-immune microenvironments (FDR-adjusted *p* < 0.05) were tested against the set of genes defining the PDL1 pathway from the Kyoto Encyclopedia of Genes and Genomes (KEGG) database. Since LAG3 was a notable feature in the liver immune TME, we also used GeneMania [17] to define a network of genes associated with *Lag3* and *Fgl1*, a ligand of LAG3 predominantly found in the liver [18]. Our analysis revealed that both the PDL1 signaling pathway (Additional file 1: Figure S7) and FGL1-LAG3 network (Additional file 1: Figure S8) are significantly enriched in the liver. Significant enrichment of the FGL1-LAG3 network in the liver non-immune microenvironment was consistent with our cytometric analysis of the immune cells showing distinctive presence of LAG3^+^ T and NK cells in the liver. For an unbiased exploration, we also tested the differentially expressed genes against all signaling pathways in the KEGG database, revealing five other enriched pathways (PPAR, Wnt, p53, ErbB, Neurotrophin), three of which (PPAR, Wnt, Neurotrophin) could be attributed to the intrinsic parenchymal differences (Additional file 1: Figure S9).

**Fig. 4:**
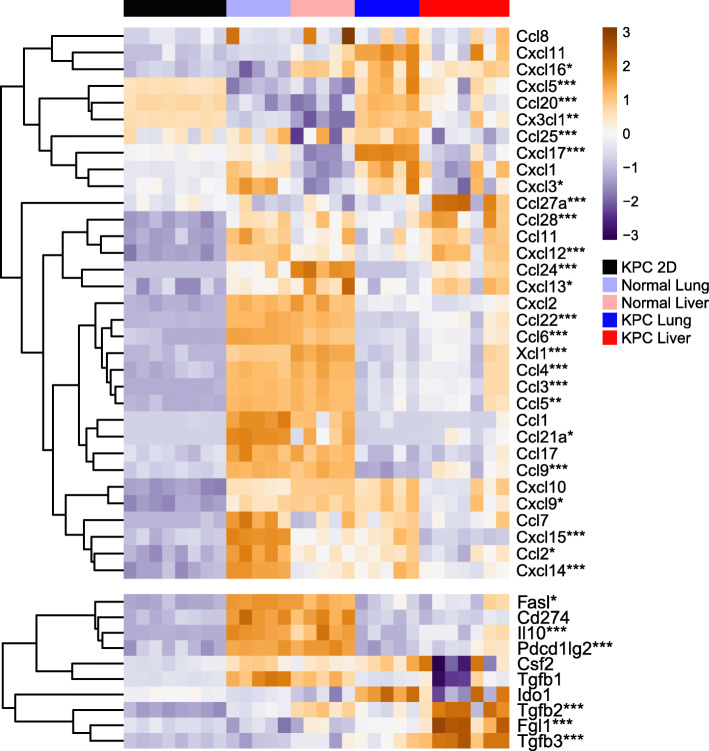
Heatmap of expression profiles for a select set of chemokine and immune regulatory genes shown for every sample. All gene expression differential analyses were performed based on the negative binomial distribution using DESeq2. FDR-adjusted *p* values *< 0.05, **< 0.01, and ***< 0.005 for comparison between the liver and lung TME sites are shown next to each gene name

In addition, to further interrogate the possible points of interplay between the immune and non-immune compartments of the metastatic microenvironment, we also focused our analysis on a select set of chemokines [19] and a set of immune markers [20] known to regulate immune function. Chemokines associated with pro-immune, immune-recruiting functions (*Cxcl9*, *Cxcl10*, *Cxcl11*, *Cxcl14*) were expressed at higher levels in the lung than in the liver (Fig. 4 and Additional file 1: Figure S10A). For *Ccl5*, *Ccl22*, *Ccl27a*, *Ccl28*, and *Cxcl12*, chemokines known to exert pro-tumor effects, substantially higher levels were noted in the liver. Among the immune regulatory molecules compared, *Pdcd1lg2*, *Tgfb2*, *Tgfb3*, *Fasl*, *IL10*, and *Fgl1* were all expressed at higher levels in the liver than in the lung (Fig. 4 and Additional file 1: Figure S10B). These findings suggest that the non-immune compartment of the liver metastatic microenvironment creates a relatively immune-suppressed environment compared to that of the lung.

To characterize few key immune regulatory chemokines in more depth, namely CCL27/CCL28 (which attract Tregs via CCR10 [21, 22]) and CXCL12 (which attract immunosuppressive myeloid cells into the microenvironment via CXCR4 [23, 24]), and to validate the immune profiles from our KPC model in additional KPC models, we performed CyTOF again with a revised panel of antibodies spanning 40+ channels (Additional file 5: Table S4) that includes CCL27 and CXCL12 along with PDX1, pan-keratin, and podoplanin (for non-immune cells). In this run, sample barcodes were based on a combination of CD29 (integrin beta-1), CD98 (large neutral amino acid transporter), and CD45 to broaden the types of cells being examined. With a total of 33 annotated cell type clusters (Additional file 6: Table S5), we performed a side-by-side comparison of the metastatic tissue profiles from mice bearing our KPC, the 2838c3 KPC (“KPCc3”), or the 6419c5 KPC (“KPCc5”) tumors [25] along with normal controls. Again, across all models, we observed that T cells and dendritic cells were overall much more abundant in the lung, whereas B cells were more abundant in the liver (Additional file 1: Figure S11A). When comparing specific immune cell subtypes that are tumor-associated, i.e., significantly higher in proportion than in the normal controls, immunosuppressive myeloid cells and LAG3-high effector T cell populations were consistently more prevalent in the liver metastatic microenvironment (Additional file 1: Figure S11B). Expression of CCL27 and CXCL12 chemokines were highest in subpopulations of KPC cells (PDX1^+^, pan-keratin^+^; “KPC_II”, “KPC_III”; Additional file 1: Figure S11C) which were greater in proportion in the liver samples compared to the lung samples, corroborating the RNAseq data.

### Human rapid autopsy specimens recapitulate immune signaling differences observed in mouse models

To correlate the findings from the KPC mouse model with human disease, five matched liver and lung metastatic samples from deceased PDAC patients were identified in the Johns Hopkins Rapid Autopsy Biobank. Sample sources and their general treatment histories are tabulated separately (Additional file 5: Table S4). Immunohistochemical staining of canonical immune markers, CD4, CD8, CD20, and CD68, were first performed and compared between the two organ sites. Similar to what was observed in the mouse models, the intratumoral density of CD4^+^ and CD8^+^ T cells was significantly greater at the pulmonary metastatic site (Additional file 1: Figure S12A-C). The intratumoral density of CD20^+^ B cells was also higher in the lung, consistent with mouse IHC analysis. Notably, when comparing the markers on a per-tissue block basis, there were not only inter-patient variability and inter-site variability, but also intra-site variability (Additional file 1: Figure S12D). Regarding checkpoint markers, PD1 and LAG3 expression were assessed by positive expression of each marker per T cell density (Additional file 1: Figure S12A, E). High PD1 expression was detected in both liver and lung samples without a clear pattern. LAG3 expression was also variable, but there was an observable trend, in which the highest expressing samples were from the liver, which was also observed in the mouse CyTOF data. Overall, the findings in the matched human samples were consistent with what were observed in the mouse model.

### Site-specific microenvironment features are detectable in primary tumors of lung and liver

Finally, to test the hypothesis that the site-specific features associated with metastatic pancreatic lesions stem from the intrinsic tissue microenvironment, tumor-associated and TME-associated characteristics were compared between lung and liver primary cancers using the Cancer Genome Atlas (TCGA) database. To first determine whether the molecular profiles in the lung and liver metastatic sites based on the KPC mouse model were preserved in the lung and liver TCGA datasets, we employed our transfer learning algorithm projectR [26] to project the principal component dimensions derived from the gene expression data for the cancer cells isolated in the KPC mouse metastatic model (Fig. 3b) onto TCGA datasets. Upon projecting onto cancer and adjacent normal samples from primary liver, lung, and pancreas TCGA datasets, we again observed that while PC1 explained the differences between the normal and tumor samples, PC2 captured the tissue sites differentially (Additional file 1: Figure S13A). This indicated that tumor-associated and tissue-associated signals were separately and consistently relatable across different species and primary tumor types.

To delineate whether this site-specific signal was at least in part tumor-driven and not solely parenchymal/stromal, we explored the MetMap dataset [11], a publicly available dataset based on RNAseq profiling of uniquely barcoded breast cancer cell lines injected in vivo for spread onto five different metastatic sites. Because these are barcoded cell lines, the dataset specifically represents cancer cell-intrinsic differences. Based on PC analysis, we found that PC1 is most highly represented by the cancer cells within the liver (Additional file 1: Figure S13B). Then, upon projecting PC1 from MetMap onto our KPC dataset, we found that PC1 was much more strongly recapitulated by the KPC samples from liver (Additional file 1: Figure S13C), suggesting that the lung and liver metastatic sites also differentially shapes tumor-intrinsic features.

**Fig. 5:**
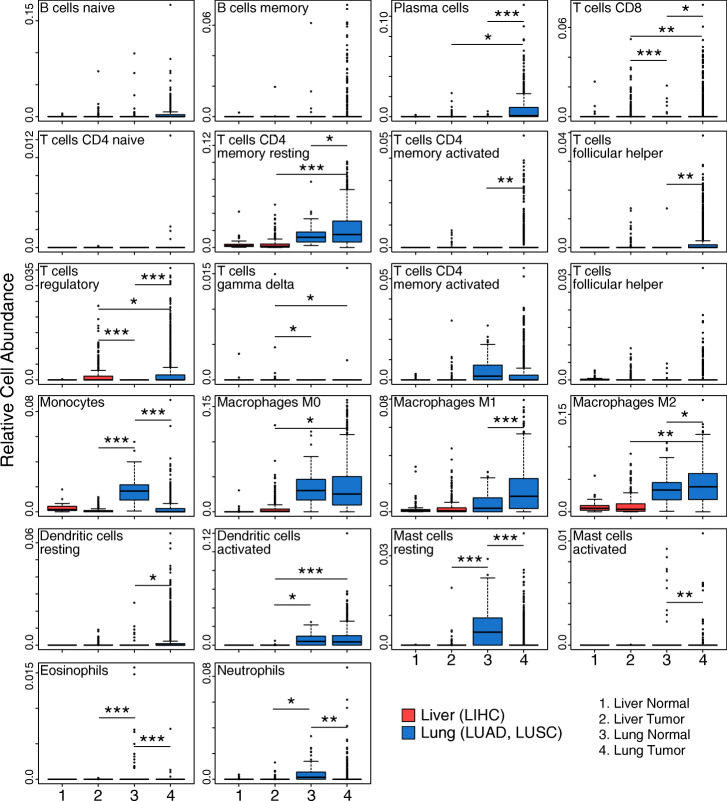
Immune cell types in lung and liver TCGA samples deconvolved by MIXTURE algorithm. Both tumor and adjacent normal samples in the TCGA databases for liver hepatocellular carcinoma (LIHC; normal *n* = 50, tumor *n* = 366) and lung adenocarcinoma and squamous cell carcinomas (LUAD and LUSC; normal *n* = 105, tumor *n* = 992) are assessed for computed relative abundances of immune cell types (coefficients). Summarizing boxplots are shown. FDR-adjusted *p* values for Tukey HSD *< 0.1, **< 0.01, and ***< 0.001

**Fig. 6:**
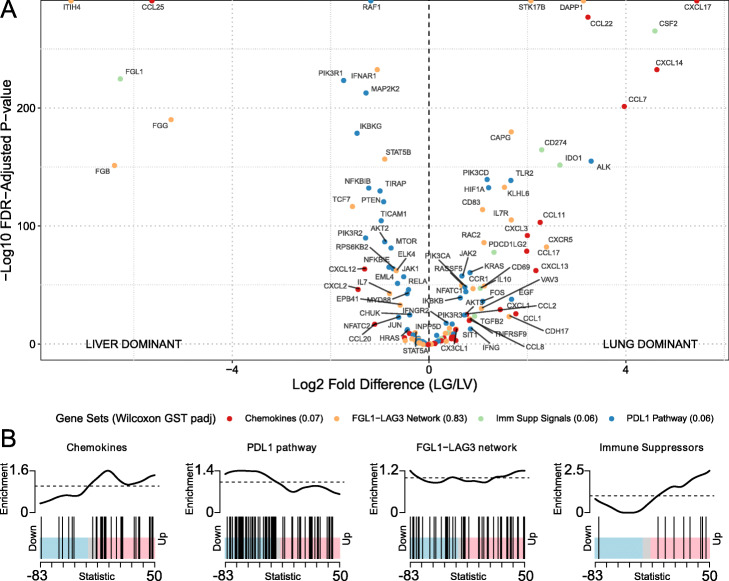
Differential expression of select gene sets comparing lung and liver TCGA datasets. **a** Volcano plot of − log10 FDR-adjusted *p* values by *DESeq2* are shown over log2-fold differences between lung and liver sites for four sets of genes (as analyzed for mouse and human metastatic pancreatic cancer samples). Gene set analysis was performed using the Wilcoxon Gene Set Test (GST) (FDR-adjusted *p* values noted). **b** Barcode plots representing the results of the gene set enrichment analyses

Next, to discern what tissue-specific features were being preserved, we also compared the TME-related features in the lung and liver TCGA datasets. Based on MIXTURE deconvolution [27] of the RNAseq datasets, lung cancers demonstrated significantly higher levels of immune cells, including CD8^+^ T cells, CD4^+^ T cells, Tregs, dendritic cells, and macrophages (Fig. 5). The differences were also present in the adjacent normal tissues, suggesting that the features are driven by the tissue microenvironment. Importantly, these findings were consistent with the pancreatic cancer mouse model and rapid autopsy analyses. Similarly, differential expression of select genes involved in immune regulation of cancers as analyzed in the mouse models and the human datasets for pancreatic metastatic disease were compared between the lung and liver primary cancers. Again, many of the immune-modulatory genes differentially expressed in the mouse model were site-specifically recapitulated, e.g., higher *CCL2*, *CXCL5*, *CXCL9*, *CXCL11*, *CXCL14*, *CXCL17*, and *IDO1* in lung cancers and higher *CXCL12*, *FGL1*, and *LAG3* in liver cancers (Fig. 6a). Gene set analysis showed that the chemokine, PDL1 pathway, and immune regulatory gene lists were highly enriched (FDR *p* values < 0.1, Fig. 6b). Several genes were also, however, inconsistent with the mouse model data including *CCL5*, *CCL20*, *CCL22*, *CCL28*, *CXCL10*, and *PDL2*, suggesting that these features may not be as robustly tissue-specific across different primary cancers. Taken together, these results strongly support the concept that many of the TME signatures in metastatic sites can be explained by the features intrinsic to the tissue. Furthermore, consistent with our hypothesis, the presence of baseline differences in the non-tumor-bearing tissues is particularly apparent. Our analysis suggests that for a given tissue site, particular pathways are involved similarly in modulating — suppressing or stimulating — the immune response to different tumor types. In all of the datasets analyzed, liver exhibited a relatively greater presence of features that characterize an immune-suppressive microenvironment compared to lung.

## Discussion

To our knowledge, our study is the first to reveal site-specific differences in the TME and adjacent normal tissue between hepatic and pulmonary PDAC metastasis. We demonstrated that the lung TME displays higher levels of immune activation and infiltration, whereas immunoregulatory networks are enriched in the liver that suggest a higher threshold of immune suppression to be overcome. Importantly, our data also suggest that the greater presence of immune cells in the lung may be due to the pro-immune signals, i.e., specific chemokines, generated from the non-immune compartment in the lung. These observations are consistent with the results of an independent immune landscape analysis of TCGA demonstrating relatively higher leukocyte fractions in primary lung cancers than in hepatocellular carcinoma as well as greater lymphocyte-depleted immune signatures within the liver samples [28]. We also demonstrated that these site-specific features are not only comparable in both mouse and human datasets of metastatic PDAC, but also preserved in primary tumors of the lung and the liver, illustrating the tissue-intrinsic property of such signals.

Our results have multiple translational implications for the development of immunotherapy for PDAC, and potentially other metastatic cancers. Although immune checkpoint inhibitors have thus far failed as single agents to demonstrate clinical benefit for patients with unselected PDAC [3], our results suggest the potential for benefit for the small subset of patients with lung-only metastasis who may have a more permissive TME for systemic immunotherapy. For the larger subset of patients with liver PDAC metastases, our observation that there are increased LAG3^+^ T cells and an emphasis of LAG3-FGL1 network in liver metastasis provide an initial rationale for studying LAG3 inhibitors in combination with anti-PD1 therapy for patients with liver metastases. An emphasis on LAG3-FGL1 signaling in the liver TME is not entirely unexpected since FGL1 is predominantly expressed in the liver, and FGL1 is a ligand for LAG3 [18]. In addition, high expression of CXCL12, an important immune-suppressive chemokine in PDAC TME [29], was consistently and primarily found in the liver in both mouse and human datasets. This is in line with a prior observation that CXCL12-CXCR4 axis attenuates immunologic responses and that blockade of this axis in mouse models of liver metastases enhanced susceptibility to anti-PD-L1 therapy [29]. Anti-LAG3 antibodies and CXCR4-targeted therapies are currently being investigated in clinical trials (NCT03470922, NCT02907099, NCT04177810), and our study provides additional rationale to develop these therapeutic strategies, particularly for cancers that arise from or metastasize to the liver.

Our data in PDAC builds on prior work in various cancer models where differences in the tumor and TME were also metastatic site dependent [9]. When comparing subcutaneous and orthotopic sites, pancreatic cell lines (CD18/HPAF, FG, L3.3, L3.6pl, and BxPC3), renal cell lines (SN12C, SN12PM6), and a prostate cell line (PC-3M) develop distinctive RNA expression profiles depending on the site [30–34]. Additionally, ^1^H NMR spectroscopy identified metabolomic changes within pancreatic cell lines (Panc-1, PxPC-3) between subcutaneous and orthotopic sites [35]. Melanoma cell lines (A375P, A375SM) also had differing IL-8 cytokine production when growing subcutaneously, in the lung, or in the liver [36]. In the TME, immune cell composition was different depending on whether breast cancer cells (4T1) were implanted subcutaneously or intratibially [37]. Furthermore, not only did the immune constituents differ between subcutaneous and orthotopic sites, orthotopic CT26 tumors responded better to checkpoint blockade [38]. In fact, another study utilizing Renca renal tumor cells, by cross-implanting tumor cells harvested from one site into another, demonstrated that the site of disease is what determines the tumor response to immunotherapy [39]. These findings highlight the importance of understanding the site-specific features of the TME.

Building on previous work using transfer learning for cross-species analysis in developmental biology [40, 41], our application of transfer learning to the RNAseq data enables us to quantify conserved biology between mouse and human from the different sites. Specifically, projecting of gene signatures between our mouse model, TCGA, and MetMap datasets enables in silico validation of the separation of both disease-related and tissue-intrinsic differences observed in the purified tumor cells from our mouse model. Moreover, our separate analysis of the immune microenvironment demonstrated that there are significant differences in the TME between liver and lung but also tissue-intrinsic differences that are present in the resident tissues even before tumors are established. This observation is expected since each organ has a unique function and requires a set of cell types to carry out its proper function. This is also interesting from the standpoint of TME-targeted therapeutic approaches. For example, one could hypothesize that as long as the target is exploited to take on an antitumor role within that tissue microenvironment, the same approach may be rational for cancers in a particular anatomical region irrespective of the cancer type. In fact, the success of checkpoint inhibitors against both primary and metastatic upper aerodigestive malignancies may be a prime example of the importance of the site of the TME. This concept suggests that tumor site may be another key factor that determines clinical outcomes of checkpoint immunotherapy, adding to other known factors such as stromal features, tumor mutational burden, and the quality of the tumor antigens. Moreover, in addition to enabling cross-species analysis, our transfer learning approach may enable cross-tissue analysis to further resolve tissues-specific TME and tumor cell features, providing a powerful tool for translating findings from primary tumors to identifying targets of cancer metastasis in a rapid and scalable way.

The experimental methodology that we have used to interrogate the biology of tissue-specific metastasis, i.e., injecting the same cell line into two different locations to generate distinct metastatic sites, has both strengths and limitations. The strength of this approach from a biological perspective is that the genetic starting point of the cancer cells for both metastatic sites is identical and enables the interpretation of meaningful differences as a result of the cancer’s interaction with the TME. This approach also overcomes the challenges that are associated with sporadic metastasis models such as establishing comparable mouse cohorts at a particular timepoint while controlling for unpredictable confounding factors. A limitation of our methodology is that our controlled implantations into distinct organs do not fully recapitulate the complexity of the metastasis process from one site to another, including clonal evolution. We were able to mitigate this uncertainty in part by extending our findings to human metastatic autopsy samples. Given the limited availability of matched metastatic samples, further work on corroborating the clinical relevance of these findings using a larger cohort of human clinical specimens would be challenging but clearly valuable. In addition to using a larger cohort, employing other types of assays would empower deeper understanding of the metastatic TME. Future work leveraging single-cell RNAseq could enhance our inference between the immune and non-immune compartments within the metastatic TME by enabling ligand-receptor analysis [42–44]. These computational inferences would be further enhanced through emerging high-dimensional spatial molecular technologies [45–47] or multi-omics technologies for simultaneous TCR profiling. Thus, such approaches would overcome some of the technical limitations in this study and yield insights beyond what could be attained solely through bulk RNAseq, CyTOF, or single stain IHC employed in this current study.

In summary, our study delineates tissue specificity of immune-modulatory pathways in metastatic PDAC TMEs. Importantly, we found that the liver TME harbored lower infiltration of activated T cells in association with emphasis of key immunosuppressive mechanisms including LAG3-FGL1 and CXCR4-CXCL12 pathways compared to the lung TME. These findings provide a basis for how the metastatic TME may differentially impact clinical outcomes and suggest the importance of developing therapeutic strategies accordingly. Our observations provide additional rationale for building on previous studies [18, 24, 29] to further investigate LAG3-FGL1 and CXCR4-CXCL12 targeting strategies especially in the setting of liver metastatic cancers. Such efforts may help to overcome the poor prognosis conferred by hepatic involvement in pancreatic cancers as well as other cancers.

## Materials and methods

### Cell lines

KrasLSL.G12D/+;p53R172H/+;PdxCretg/+ (KPC) is a pancreatic cancer cell line driven by Kras and TP53 mutations under the pancreas-specific pdx-1 promoter in C57Bl/6 background [12, 13]. KPC 2838c3 and KPC 6419c5 cell lines were purchased from Kerafast. They are maintained in RPMI containing glutamine, 10% FBS, 0.1 mM non-essential amino acids, 1 mM sodium pyruvate, and 1X penicillin/streptomycin in 5% CO_2_.

### Animals

Female C57Bl6 mice at 6–8 weeks of age were purchased from Jackson Laboratories and maintained in accordance with the Institutional Animal Care and Use Committee (IACUC) guidelines. The hemispleen technique of establishing a hepatic metastatic model of pancreatic cancer by intraportal injection has been previously described [16]. Briefly, a left subcostal incision is made to reveal the spleen, which is clipped and hemisected. One half of the spleen is used to inoculate 3.5 × 10^5^ KPC cells in 100 μl volume and flushed with 150 μl PBS in the same injection. Slow injection allows for visualization of intraportal flushing, after which the splenic vessels are clipped and the injection portion is removed. To establish a pulmonary metastatic model of pancreatic cancer, 5 × 10^5^ KPC cells are injected intravenously in 100 μl total volume via tail vein. By day 21 from injection day, mice are euthanized in the CO_2_ chamber for harvesting the liver and lungs. Tumor burden in the liver and lung, respectively, is grossly visible and histologically confirmed by formalin fixation, paraffin embedding, and hematoxylin/eosin staining following standard procedures.

### Rapid autopsy database

The Legacy Gift Rapid Autopsy program was approved by the Johns Hopkins institutional review board and deemed in accordance with the Health Insurance Portability and Accountability Act. With informed consent, patient samples were collected via an autopsy as soon as possible after death, typically within 6 h. Harvested primary and metastatic samples are formalin fixed for paraffin embedding and assigned unique identifiers. Documentation of every sample is based on gross and microscopic examination along with the corresponding patients’ medical history.

### Immunohistochemistry

Slides were stained for immune markers following standard immunohistochemistry protocols as previously described. The following is the list of the antibodies used, source, and antigen retrieval method: Pan-cytokeratin (MS343R7, ThermoScientific, Ventana Ultra CC1 buffer), CD4 (Sp35, Ventana, EDTA pH9.0), CD8 (C8/144B, Cell Marque, EDTA pH9.0), CD20 (L26, Ventana, EDTA pH9.0), CD68 (KP-1, Ventana, Citrate pH6.0), PD1 (NAT105, Cell Marque, 1:1000, Citrate pH6.0 [48]), and Lag3 (17B4, LifeSpan BioSciences, 0.1 μg/mL, Citrate pH6.0 [49]). Quality of the staining results for every marker was reviewed by a pathologist (E.D.T.). Mouse tissues were stained with the following antibodies: CD8 (D4W2Z, Cell Signaling Technologies), CD4 (EPR19514, Abcam), B220 (RA3-6B2, Novus Bio), and CD68 (rabbit polyclonal, Abcam). Stained slides were scanned into digital formats using the Aperio ScanScope® CS system (Leica). Digital slides were annotated to demarcate the matching regions of interest and then quantitatively assessed for staining intensity, cellular density of positive staining, and area density of positive staining using HALO (Indica). For unbiased analysis, quantification module parameters were first manually optimized to analyze three matching slides of liver and lung tissues and were kept the same for all slides.

### Antibodies for cytometry

A list of CyTOF antibodies, clones, metal isotopes, and their titrations is provided in Additional file 2: Table S1. Custom conjugation of antibodies was performed using Maxpar Lanthanide Conjugation Kits (Fluidigm) according to the manufacturer’s protocol and as previously published [50]. Briefly, buffer exchange was first performed on purified antibodies using 50 kDa ultra filtration columns (Amicon), and subsequently the antibodies were partially reduced with 4 mM TCEP (Thermo Scientific). Polymers were loaded individually with isotopically enriched metals and were then conjugated onto the corresponding reduced antibodies. The conjugated antibodies were washed and quantified using Nanodrop. The final antibody concentrates were diluted in a stabilization buffer (Candor) containing 0.3% sodium azide. Each antibody was titrated by starting at the suggested dilution and titrated as needed in a range of 3–4 serial dilutions using clear positive controls, e.g., stimulated splenocytes, and validating the concentration that permits discrimination while minimizing spillover signals. For flow cytometry, the following antibodies were used: CD45 (Biolegend, clone 30-F11, FITC), CD3 (Biolegend, clone 145-2C11, Pacific Blue), CD4 (BD Biosciences, clone RM4-5, BV605), CD8 (BD Biosciences, clone 53-6.7, BV786), NK1.1 (Biolegend, clone PK136, APC), and Lag3 (Biolegend, clone C9B7W, PE).

### Mouse liver and lung sample preparation

Upon harvest, mouse lung and liver samples were enzymatically dissociated using Lung Dissociation Kit and Liver Dissociation Kit (Miltenyi) per manufacturer’s instructions. Briefly, each of the organ was incubated in an enzymatic mix on gentleMACS™ Octo Dissociator with Heaters (Miltenyi), an automated benchtop machine providing programmed cycles of slow and rapid mechanical dissociation at 37 °C. The homogenate is filtered using 100 μm cell strainers into a new 50-ml conical tube and quenched using complete RPMI media. Due to relatively high amounts of non-cellular debris in the liver, the homogenates were further cleaned using a simplified gradient centrifugation using percoll (GE Healthcare) 40% underlaid with 80%, allowing for exclusion of dead cells, debris, or red blood cells. After a second wash with complete RPMI, pellets made of single cells were obtained. Greater than ninety percent viability was confirmed using Trypan Blue.

### CyTOF staining and preprocessing

Approximately 1.5 million cells per sample were plated onto a 96-well plate and all samples were rinsed with PBS with 2 mM EDTA after plating. Live/dead staining was performed with 5-min incubation in cis-platinum solution (Fluidigm), subsequently quenched with complete media. For multiplexing samples, a modified live cell barcoding strategy was used [51], incorporating four different metals conjugated to CD45 antibodies for utilizing 4-plex batching scheme (Supplementary Table 1). Each sample was then stained with a unique barcode for 25 min at room temperature in cell staining buffer (CSB, Fluidigm). Batched samples were then combined into a single tube and washed. Multiplexed samples were then incubated in Fc block (BD Biosciences) for 10 min at room temperature. Surface staining was first done by incubating the tube in a cocktail of surface marker antibodies for 30 min at room temperature. After two washes, intracellular staining was performed using Foxp3 Transcription Factor Fix/Perm kit (Thermo Fisher) per manufacturer’s protocol, fixing for 30 min followed by staining for 30 min at room temperature. Upon completion of staining, cells were stored in fresh 1% methanol-free formaldehyde in PBS (Thermo Scientific) until the day of data collection. Just before data collection, all cells were labeled with Iridium (Fluidigm) at 1:3000 in Maxpar Fix/Perm Buffer (Fluidigm) for 30 min at room temperature. All events were acquired on a CyTOF1 mass cytometer (Fluidigm). Mass cytometry data were acquired at the University of Maryland School of Medicine Center for Innovative Biomedical Resources (CIBR) Flow Cytometry and Mass Cytometry Core Facility, Baltimore, Maryland. Randomization, bead normalization, and bead removal of data collected were performed on CyTOF software (Fluidigm) v6.7. Using FlowJo (BD) v10.5, single-cell events were identified by gating on a tight population based on cell length and Iridium signal. Dead cells were then eliminated by manually gating out cells positive for 194/196Pt on a biaxial plot. Debarcoding was carried out by manual gating to select for events that are positive for each of the four CD45 metals assigned to the sample and negative for the remaining three CD45 metals. Compensation of batch effects related to the live cell barcodes was performed via *CATALYST* software [52] by first computing the spillover matrices unique to each individual barcode based on singly stained splenocyte samples and applying the compensation matrices onto the samples.

### Flow cytometry

Prepared single-cell suspensions were first blocked using Fc block for 10 min followed by the addition of the following cocktail of antibodies for 30 min at room temperature: CD45 (1:100), CD3 (1:100), CD4 (1:100), CD8 (1:100), NK1.1 (1:100), and Lag3 (1:100). For fluorescence spillover compensation, single stain and negative stain controls were used. Cells were washed and run on CytoFLEX (Beckman Coulter) for data acquisition. Data analysis was performed using FlowJo (BD) v10.5.

### Whole exome sequencing

Raw BCL files generated by the sequencer were converted to fastq files for each sample using bcl2fastq v.2.19. Raw sequencing data are evaluated with FastQC, a tool for assessing sequencing quality. Sequence reads were trimmed to remove possible adapter sequences and nucleotides with poor quality using Trimmomatic v.0.38. Cleaned reads were then aligned to GRCm38 reference genome using Illumina Dragen Bio-IT Platform. BAM files were generated as a result of this step.

### RNAseq data acquisition and preprocessing

Five groups were prepared for RNAseq: (i) KPC-bearing liver, (ii) KPC-bearing lungs, (iii) normal liver control, (iv) normal lung control, and (v) KPC cells from in vitro culture as control. Liver and lungs were first enzymatically dissociated into single-cell samples as described above. To assess the non-immune component of the TME, KPC-bearing samples were further enriched for tumor and non-immune cells by Tumor Isolation Kit (Miltenyi) according to manufacturer’s instructions. Briefly, single-celled samples were incubated with magnetic beads targeting a mixture of antigens including, but not limited to, CD45, CD31, and Ter119, for 15 min on ice. Samples were then run through LS columns (Miltenyi) placed on MACS Multistand (Miltenyi) to negatively select for unbound cells that pass through the magnetic field. Enriched samples were collected into new tubes, washed, quantified, and pelleted. Greater than ninety percent viability of cells was confirmed during quantification using Trypan Blue. Pellets were then resuspended in RNA lysis buffer (Zymo Research) and stored at – 20 °C until the extraction step. Total RNA was extracted using Quick-RNA Microprep Kit (Zymoresearch, Cat# R1050) with in-column DNase I treatment. NEBNext Ultra II Directional RNA Library Prep Kit for Illumina was used to generate libraries. Polyadenylated RNA is first selected using oligo dT beads and fragmented. After cDNA synthesis, adapters are ligated for PCR enrichment. Unique dual indexes were applied to different samples for NovaSeq sequencing. The BioAnalyzer is used for quality control of the libraries to ensure adequate concentration and appropriate fragment size. Libraries were uniquely barcoded and pooled for sequencing. DNA sequencing was performed on an Illumina® NovaSeq6000 instrument using standard protocols for 50 bp paired end sequencing. Illumina reads were processed through Illumina’s Real-Time Analysis (RTA) software generating base calls and corresponding base call quality scores. FASTQ files were generated with Illumina’s bcl2fastq2. Resulting data was aligned to a reference mouse genome M21 using the *salmon* software [53]. Values were normalized as reads per million (RPM) and the resulting count data was log2 transformed.

### TCGA data

Gene-level RNAseq datasets were downloaded from Genomic Data Commons harmonized database using the TCGAbiolinks package [54]. All tumor and adjacent normal samples from liver hepatocellular carcinoma (LIHC), lung adenocarcinoma (LUAD), and lung squamous cell carcinoma (LUSC) were obtained. As gene expression, we used fragments per kilobase of transcript per million mapped reads upper quartile (FPKM-UQ) that were log2-transformed for further analysis.

### TCRseq analysis

TCR repertoire was assessed as previously described [55, 56]. Briefly, tissue scrolls from FFPE normal liver, normal lung, KPC-bearing liver, and KPC-bearing lung samples were sequenced using the immunoSeq assay (Adaptive Biotechnologies) at the survey sequencing level.

### Bioinformatics and statistical analysis

#### WES analysis

Sequences for both KPC and Panc02 cell lines were aligned using the GRCm38 mouse reference genome (C57/Bl6 mouse model). Variant call files were generated using the Illumina Dragen Bio-IT Platform in somatic mode and the Ensembl Variant Effect Predictor (VEP) v95 annotation tool was used to classify mutation type. Mutations included passed all quality filters and were not identified by dsSNP ID. Additionally, 310 variants were found to be shared — as defined by the same chromosome, position, and reference and alternate nucleotides — by both the KPC and Panc02 variant files and were excluded from the analysis as these were interpreted to be related to the C57Bl6 background, perhaps arising from the differences between the GRCm38 reference and the background of the two PDAC cell lines. Neoantigens were evaluated using the pVACseq program in pVACtools (v.1.5.13) in the context of the C57Bl6 mouse HLA type (class I, H-2-Kb and H-2-Db, and class II, I-Ab alleles). Class I and class II binding predictions were performed using NetMHC (v4.0) and NetMHCIIpan, respectively.

#### CyTOF analysis

A computational pipeline based on *diffcyt* [57] was employed using R v3.5. Briefly, for unsupervised clustering, FlowSOM algorithm [58] was used to identify 30 metaclusters that were then annotated into specific immune cell subtypes. Clustering was visualized using a two-dimensional uniform manifold approximation and projection (UMAP) dimensionality reduction algorithm [59]. Two thousand cells per sample were used for visualization. *edgeR* [60] was used to compare differential abundances and *limma* [61] for mean marker intensity comparisons. False discovery rate of 10% (adjusted *p* value < 0.1) were denoted in figures. For flow cytometry data, two-tailed *t* tests were used to compare the means.

#### RNAseq analysis

Differential analyses of the expression profile in KPCs, KPC-bearing liver, and KPC-bearing lung (lung vs. 2D, liver vs. 2D, lung vs. liver) were performed using the *DESeq2* package [62] based on negative binomial distribution. *p* values were FDR-adjusted with Benjamini-Hochberg method and adjusted *p* values below 0.05 were used to perform pathway analyses across all Kyoto Encyclopedia of Genes and Genomes (KEGG). Since RNAseq data represents samples without immune cells, the KEGG PD-L1 pathway, which contains two sets of genes referring to either cancer cells or T cells, was modified to subset just the set of genes related to cancer cells. PD-L2 was also added to this set of genes. To identify a set of genes that may be related to LAG3-FGL1 signaling, the two genes were browsed in GeneMania database [17] utilizing 39 co-localization, 3 physical interactions, 1 disease associations, 1 phenotype-MGI, and 1 conservation profile-phylogeny interaction networks for *M*. *musculus*. A total of 48 gene nodes were identified and used for LAG3-FGL1 pathway analysis.

#### TCGA analysis

MIXTURE algorithm [27] was used to deconvolve the bulk datasets into coefficients that represent immune cell proportions and were compared using Tukey HSD. Differential gene expression analysis was performed using *DESeq2* [62]. Gene set analysis was performed using Wilcoxon Gene Set Test.

#### TCRseq analysis

Sample clonality was calculated based on the normalized Shannon Entropy as previously described [55, 56]. Results were compared using Wilcoxon-Mann-Whitney.

## Supplementary Information

**Additional file 1: Figures S1-S13.**

**Additional file 2: Table S1.** 1^st^ Mass Cytometry Antibody Panel.

**Additional file 3: Table S2.** Mass Cytometry Major Immune Cell Annotation from 1^st^ Panel Data.

**Additional file 4: Table S3.** Mass Cytometry T Cell Subset Annotation from 1^st^ Panel Data.

**Additional file 5: Table S4.** 2^nd^ Mass Cytometry Antibody Panel.

**Additional file 6: Table S5.** Mass Cytometry Cell Subset Annotation for 2^nd^ Panel Data.

**Additional file 7: Table S6.** Rapid Autopsy Samples Additional Detail.

**Additional file 8.** Review history.

## Data Availability

The mouse datasets generated during this study are available in the Github repository, https://github.com/wonjho/KPC_Mets [63]; FlowRespository FR-FCM-Z3MW [64]; and GEO accession GSE172106 [65]. Gene-level RNAseq datasets were downloaded from Genomic Data Commons harmonized database using the TCGAbiolinks package [54]. MetMap dataset was retrieved from [11]. Sources of all publicly available databases analyzed during the current study are described in the methods section.
