# Supplementary material for: Multi-omic profiling of lung and liver tumor microenvironments of metastatic pancreatic cancer reveals site-specific immune regulatory pathways
Source: Genome Biol. 2021 May 13;22:154. doi: 10.1186/s13059-021-02363-6 (PMC8118107; doi:10.1186/s13059-021-02363-6)
Supplement: Supplementary file 1 — Additional file 1: Figures S1-S13. [file 13059_2021_2363_MOESM1_ESM.pdf]

Multi-omic profiling of lung and liver tumor microenvironments of metastatic pancreatic cancer reveals site-specific immune-regulatory pathways

Additional File 1: Figures S1-S13

Authors
Won Jin Ho[1], Rossin Erbe[1,2], Ludmila Danilova[1], Zaw Phyo[1], Emma Bigelow[1], Genevieve Stein-O'Brien[2], Dwayne L. Thomas II[1,4], Soren Charmsaz[1], Nicole Gross[1], Skylar Woolman[1], Kayla Cruz[1], Rebecca M. Munday[1,2], Neeha Zaidi[1], Todd Armstrong[1], Marcelo B. Sztein[5], Mark Yarchoan[1], Elizabeth D. Thompson[1,4], Elizabeth M. Jaffee[1,6,*], Elana J. Fertig[1,2,3,7,*]

Affiliations
[1]Department of Oncology, Sidney Kimmel Comprehensive Cancer Center, Johns Hopkins School of Medicine, Baltimore, Maryland
[2]McKusick-Nathans Institute of Genetic Medicine, Johns Hopkins University
[3]Department of Applied Mathematics and Statistics, Johns Hopkins University Whiting School of Engineering, Baltimore, MD USA
[4]Department of Pathology, Johns Hopkins University School of Medicine
[5]Center for Vaccine Development and Global Health, University of Maryland School of Medicine, Baltimore, MD, USA
[6]Skip Viragh Center for Pancreatic Cancer, Johns Hopkins University School of Medicine
[7]Department of Biomedical Engineering, Johns Hopkins University School of Medicine

*Co-corresponding authors:

Elana J. Fertig, Sidney Kimmel Comprehensive Cancer Center, Johns Hopkins University School of Medicine, 550 N Broadway Suite 1101E, Baltimore, MD 21209. Phone: 410 955 4268; E-mail: ejfertig@jhmi.edu

Elizabeth M. Jaffee, Bloomberg–Kimmel Institute for Cancer Immunotherapy, Johns Hopkins University School of Medicine, Baltimore, 4M07 Bunting Blaustein Cancer Research Building, 1650 Orleans Street, Baltimore, MD 21287. Phone: 410-955-2957; E-mail: ejaffee@jhmi.edu

Supplementary Figure 1

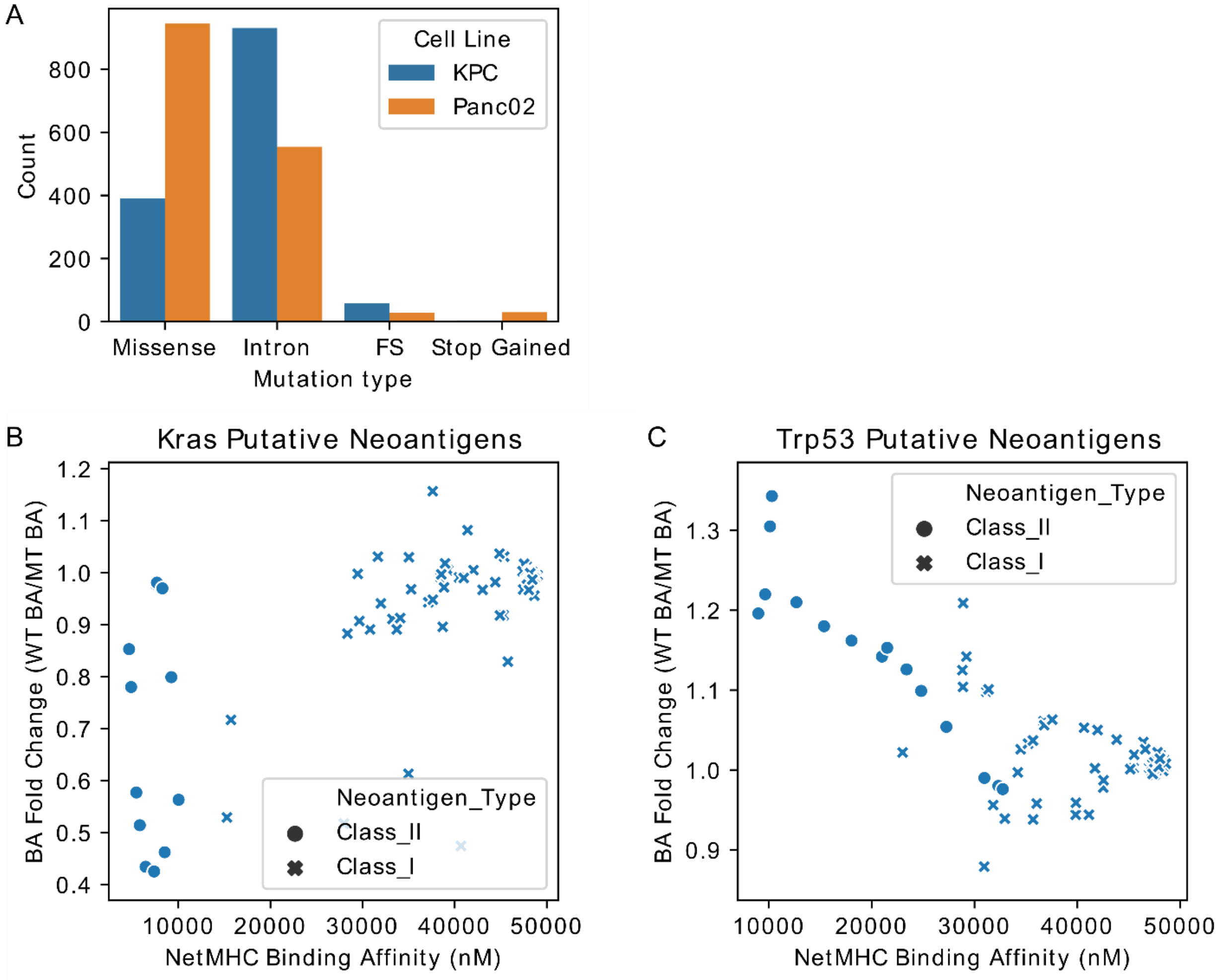

Mutational landscape of KPC cell line based on whole exome sequencing analysis. (A) Types of and numbers of each type of mutations are shown. The carcinogen-induced Panc02 cell line is used as a comparator. Class I and II neoantigens associated with the two key driver mutations in the KPC cell line, KrasG12D (B) and Trp53R172H (C) are plotted as the fold change of binding affinity (BA) of wild-type (WT) over mutant (MT) gene vs. the predicted binding affinity of each neoantigen based on NetMHC.

Supplementary Figure 2

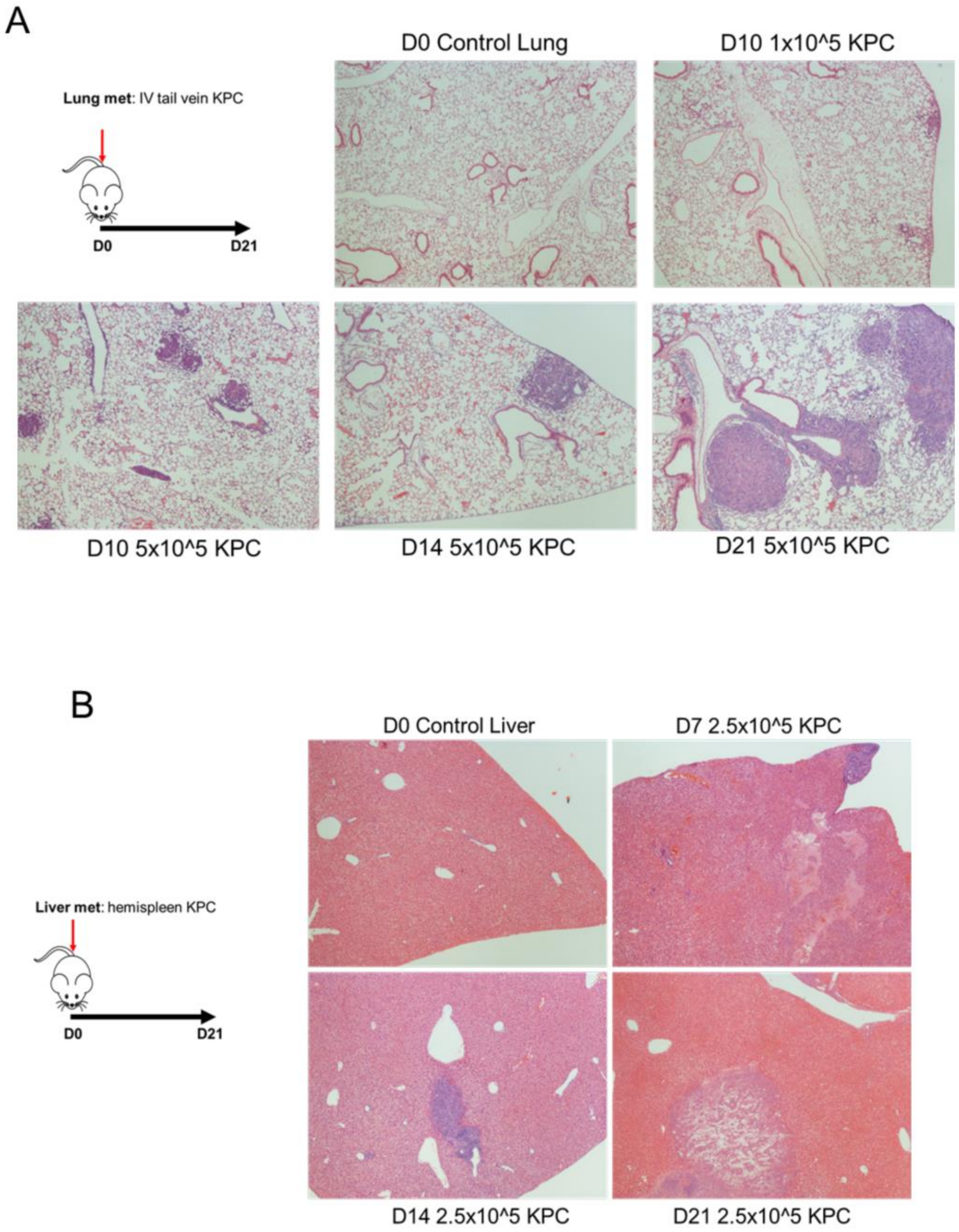

Histological confirmation of tumor burden in the metastatic models. Lung or liver metastatic model of pancreatic cancer was established with intravenous or intraportal/hemispleen injection of KPC cells on D0. By D21, robust (A) intrapulmonary and (B) intrahepatic tumor burdens were confirmed histologically.

Supplementary Figure 3

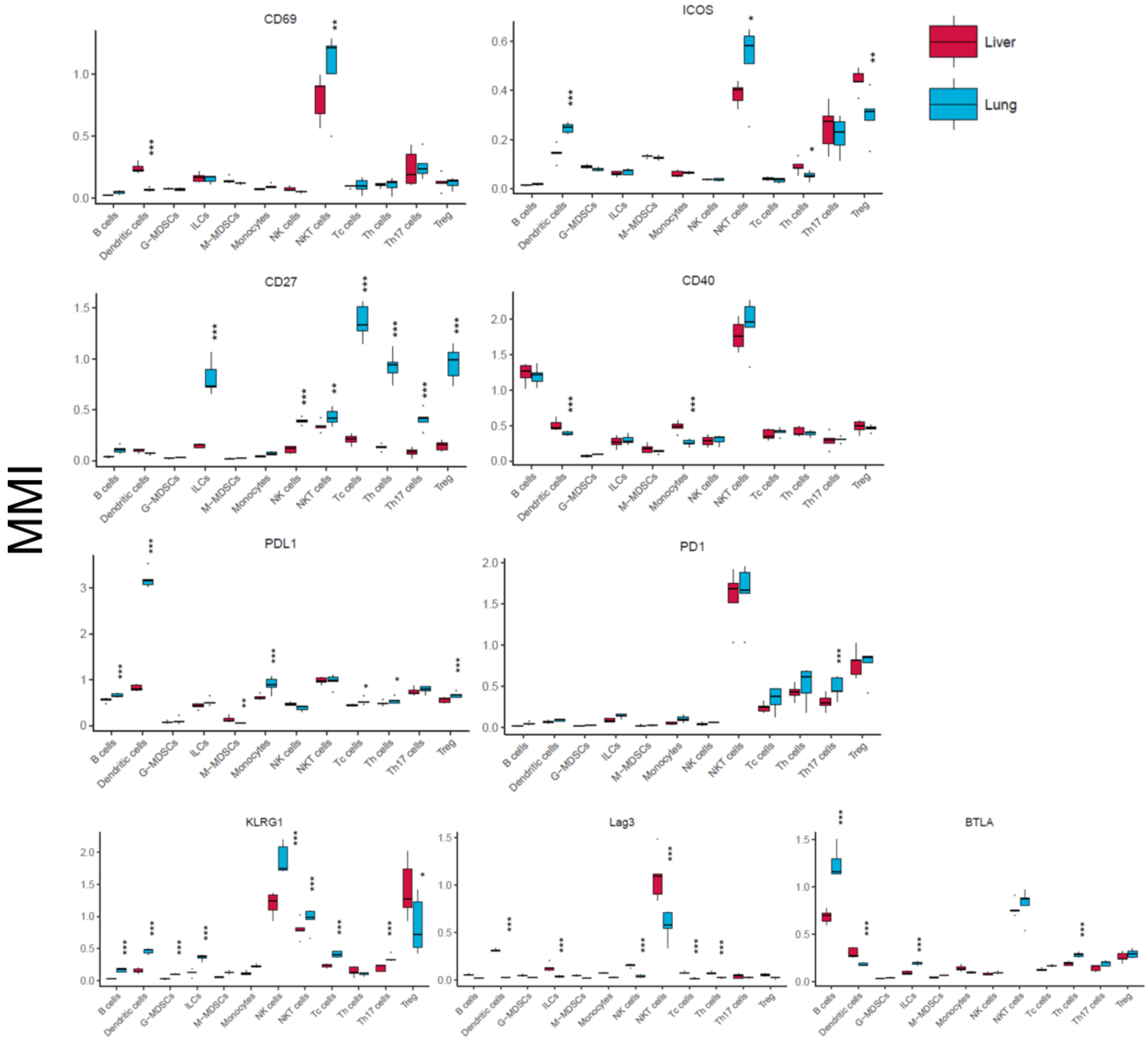

Functional profiles of immune cell clusters in KPC-bearing liver and lung TME. Mean metal intensities (MMI) of each functional marker is shown as box plots with median bars. Blue represents KPC-bearing lung and red represents KPC-bearing liver. FDR adjusted P values *<0.05, **<0.01, ***<0.005 when comparing between the two TME sites are shown. Dots represent outliers.

Supplementary Figure 4

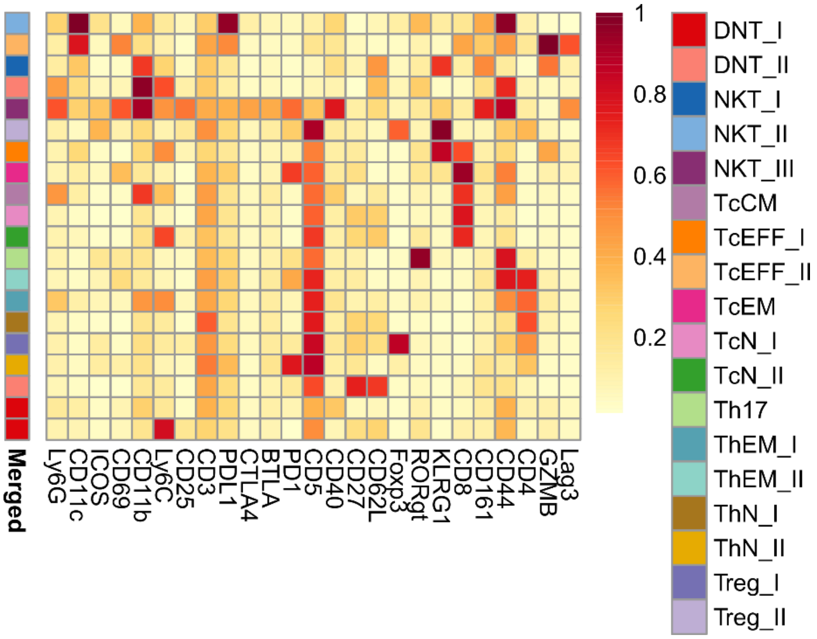

Results for FlowSOM clustering analysis using CD3+CD19-B220- cells only. Heatmap represents the normalized metal intensities of markers used for clustering (column labels) with the resulting metaclusters (rows). Color bar represents scaled expression. Final immune cell annotations of the clusters are color coded and displayed on the far left. Roman numerals indicate phenotypically distinct subtypes within the annotated cluster based on marker expressions. Abbreviations: CM, Central Memory; DNT, Double Negative T; EFF, Effector; N, Naïve; NKT, Natural Killer T; Tc, Cytotoxic T; Th, Helper T; Treg, Regulatory T.

Supplementary Figure 5

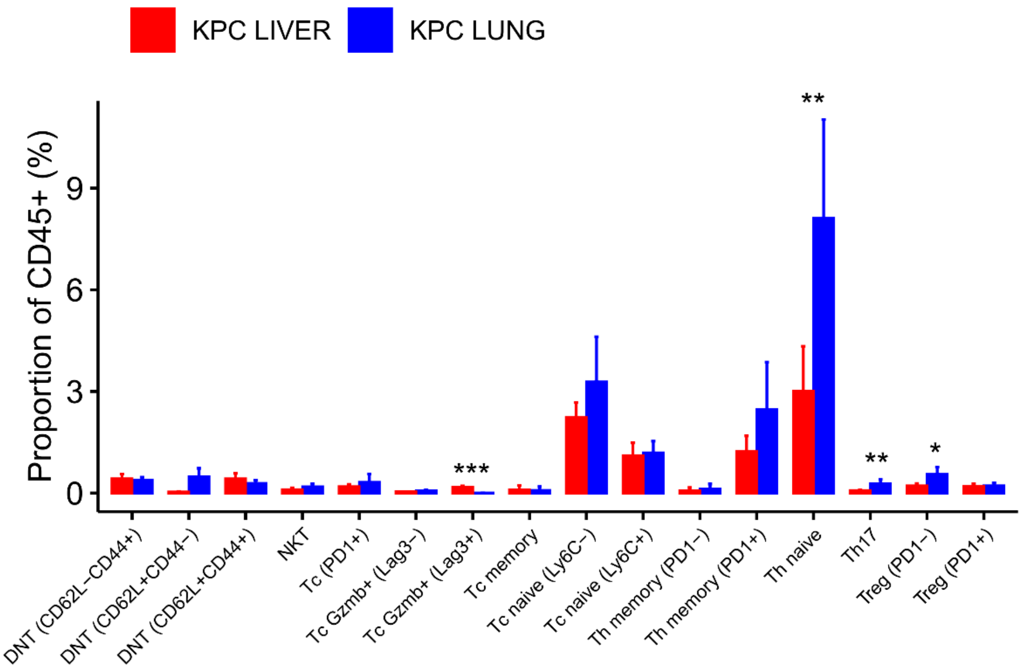

Abundance of each T cell cluster as a proportion of the entire immune compartment. All T cell clusters identified by FlowSOM are shown as a percentage of CD45+ cells in bar graphs, mean + SD. FDR adjusted P values *<0.05, **<0.01, ***<0.005 when comparing between the two TME sites are shown.

Supplementary Figure 6

A

Adjacent Normal Liver Region

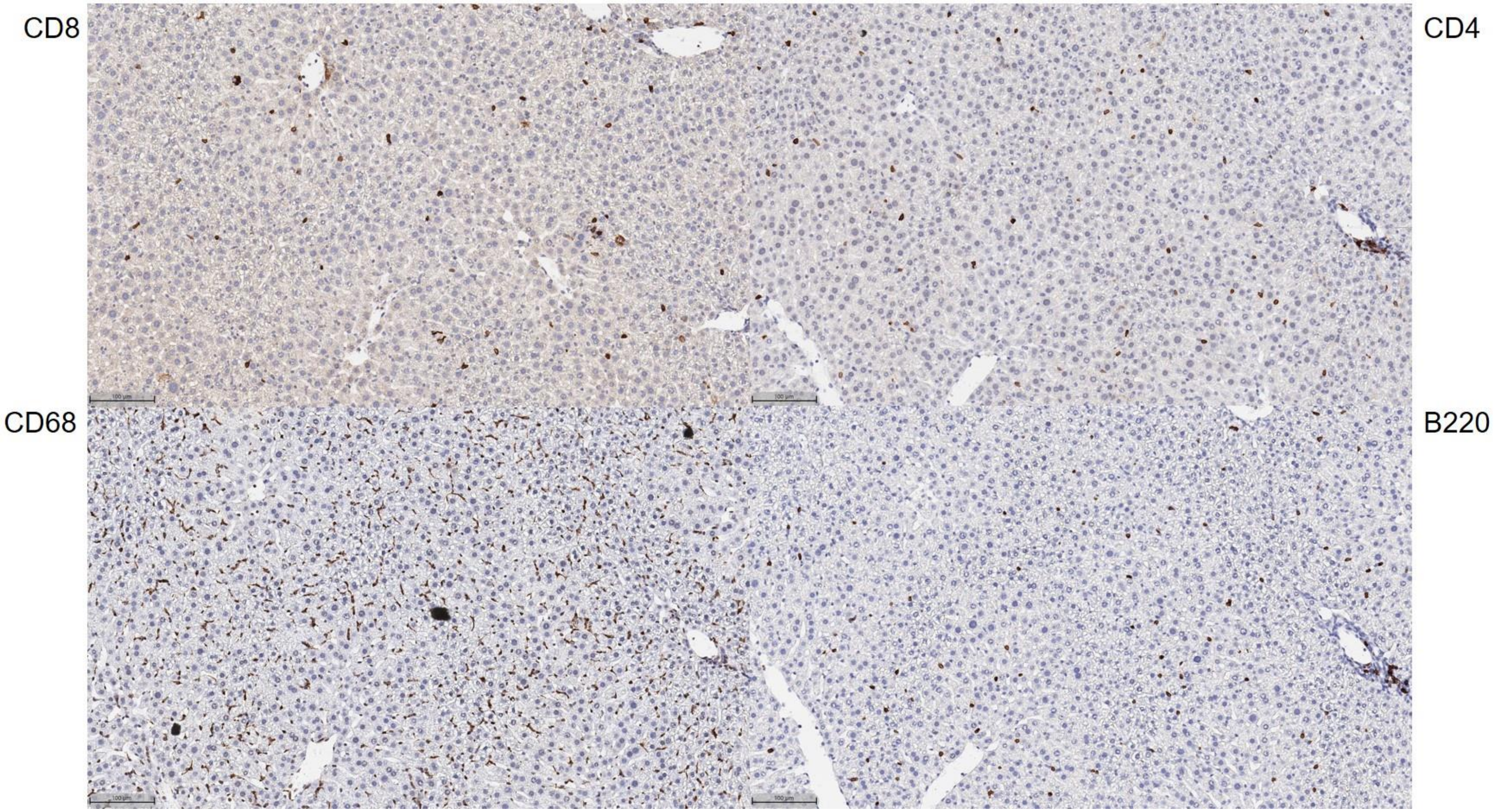

B

KPC Tumor In Liver Region 1

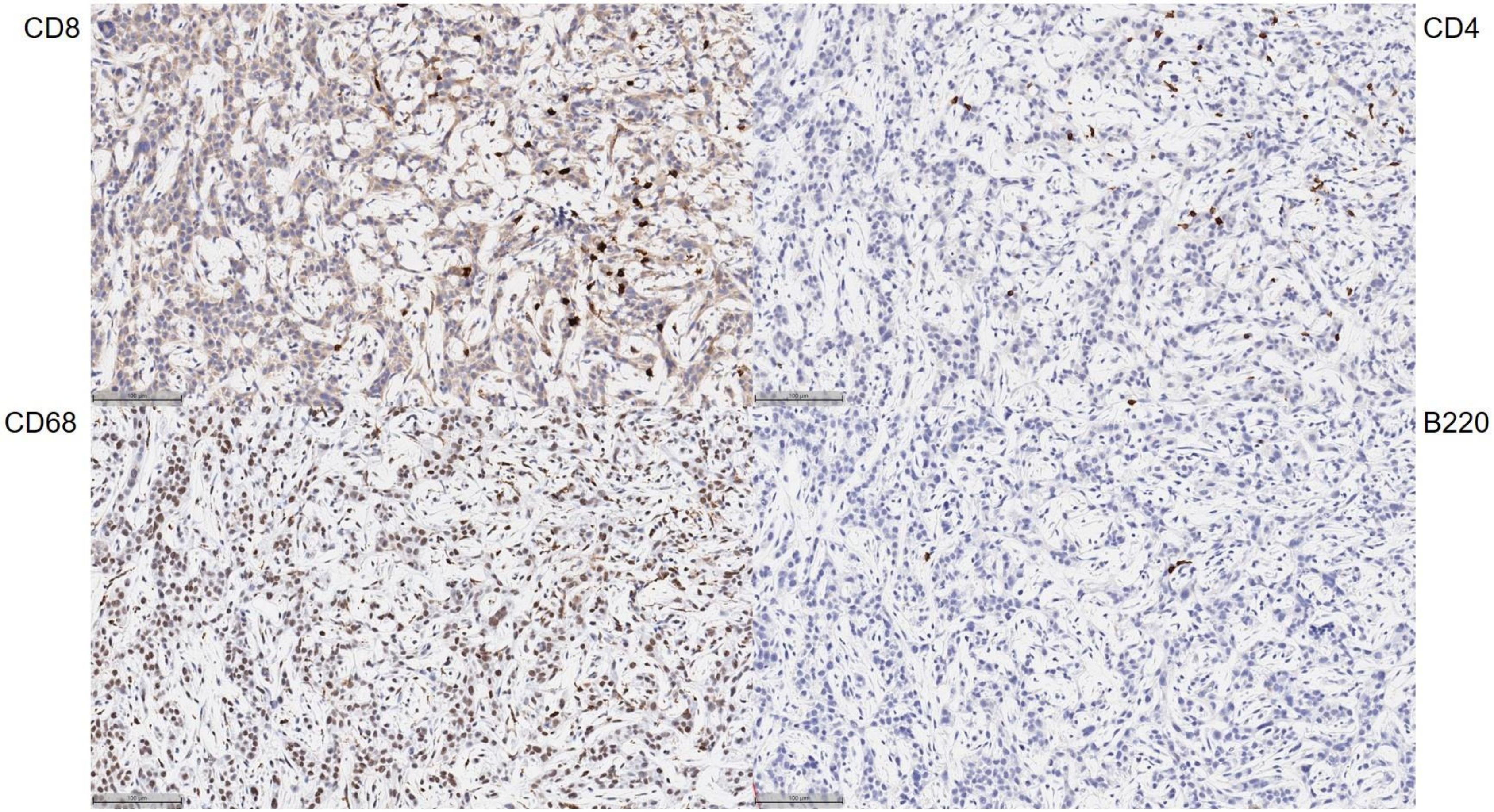

C

<u>KPC Tumor In Liver Region 2</u>

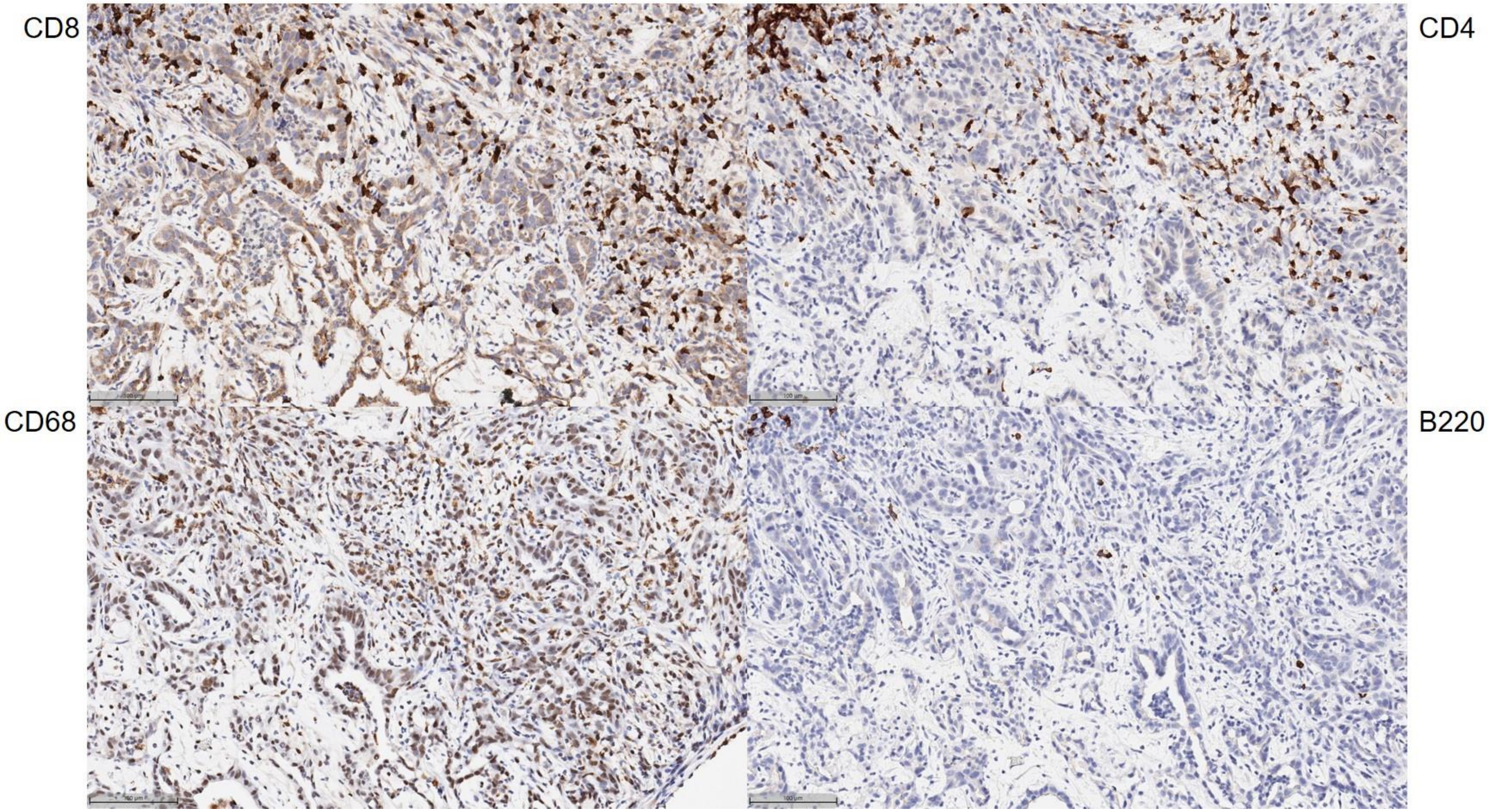

D

Adjacent Normal Lung Region

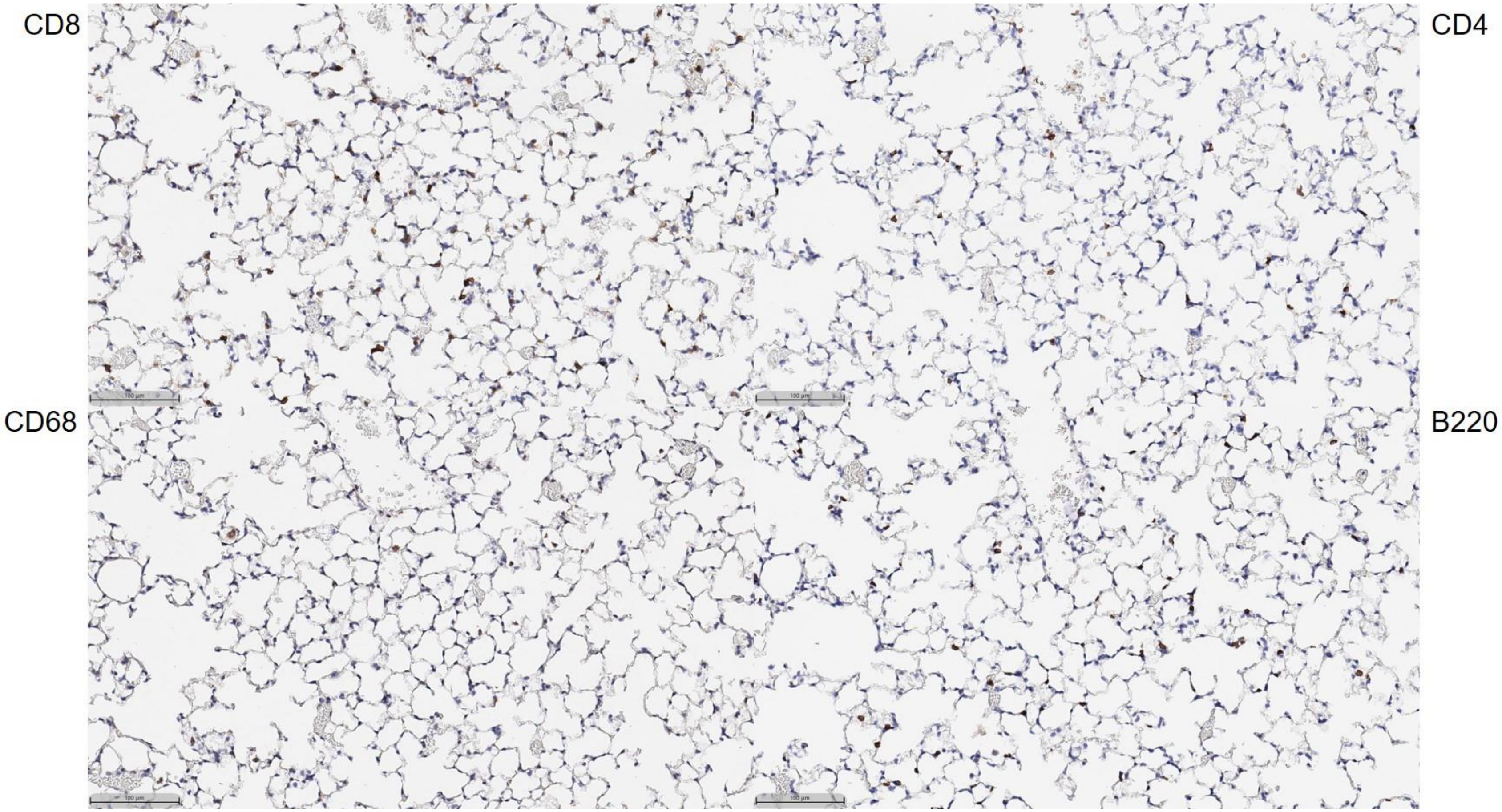

E

KPC Tumor In Lung Region 1

CD8 CD4

CD68 B220

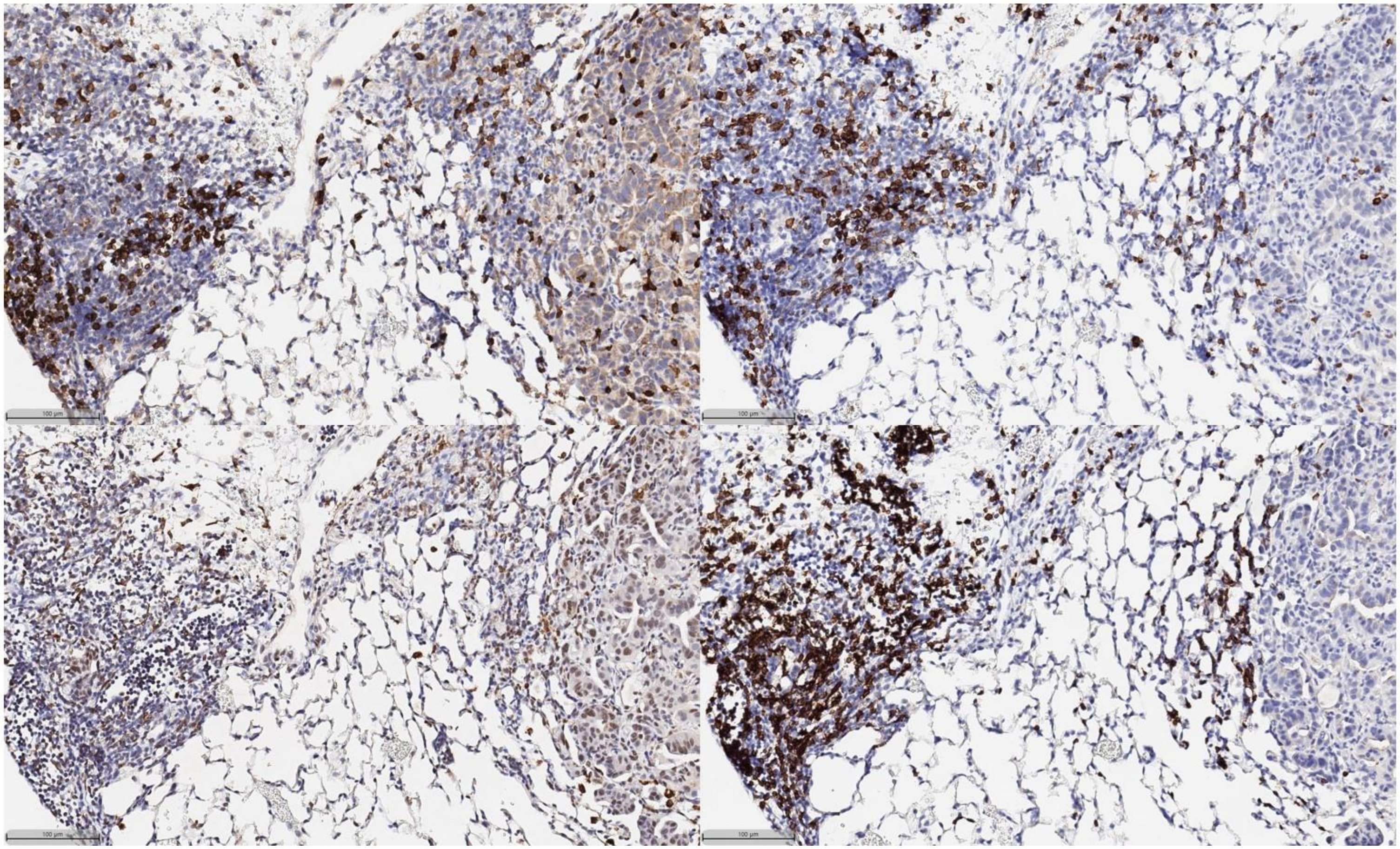

F

KPC Tumor In Lung Region 2

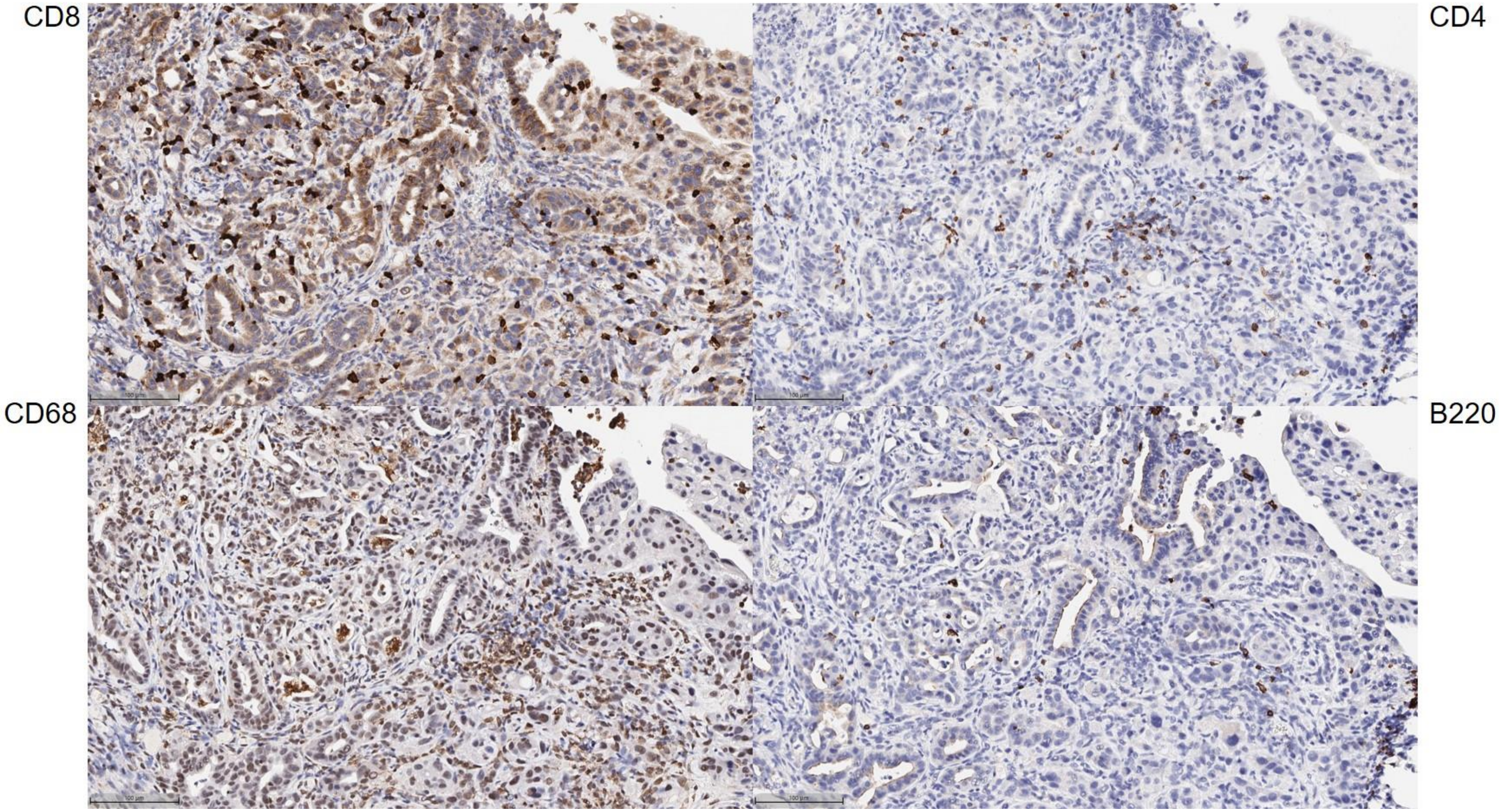

G

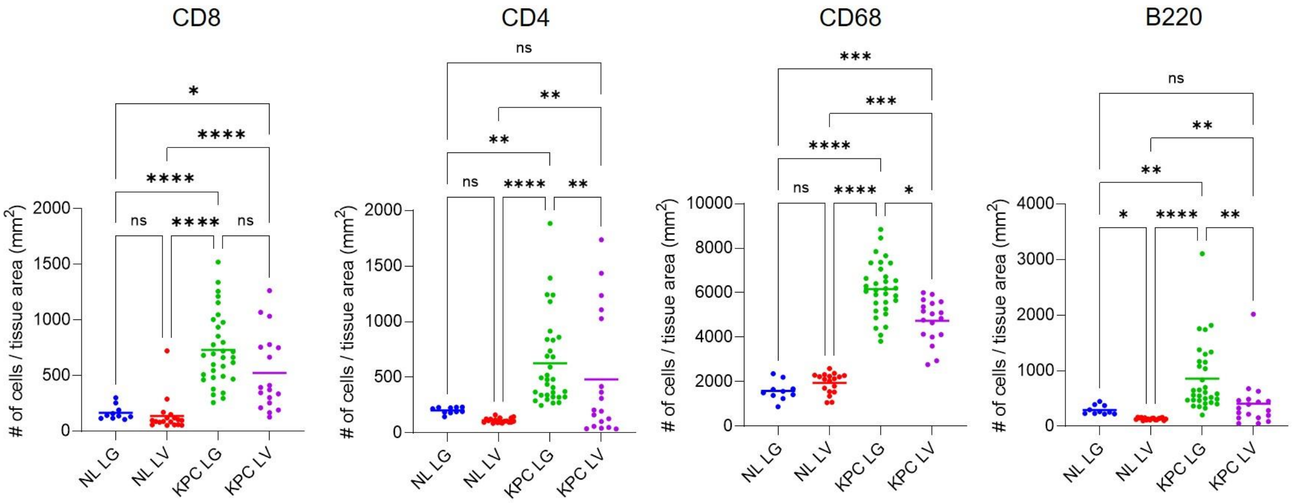

H

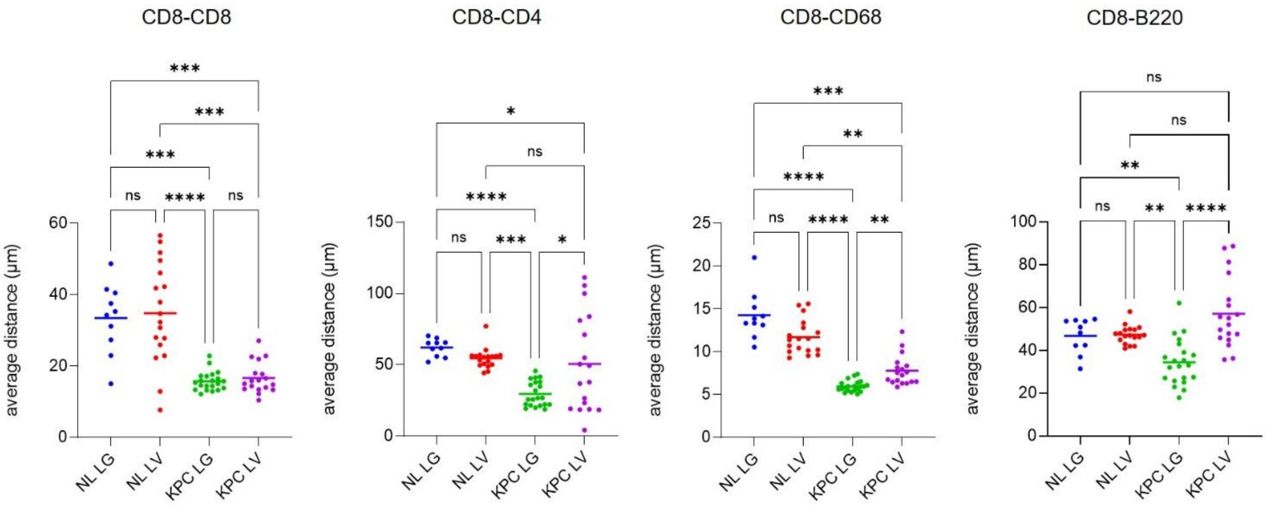

Immunohistochemistry of immune cells within liver and lung metastatic sites in the KPC mouse model. Representative IHC images for CD8 (T cells), CD4 (T cells), CD68 (macrophage/myeloid cells), and B220 (B cells) at 10X are shown (A-F), scale bar: 100µm. Adjacent normal liver (A), two intratumoral regions in the liver (B, C), adjacent normal lung (D), and two intratumoral regions are shown. (G) Density of cells positive for each marker in the KPC tumors within lungs (KPC LG) and livers (KPC LV) and the normal adjacent regions in the lungs (NL LG) and livers (NL LV) are quantified using HALO. (H) Proximity of immune cells was assessed: using HALO, adjacent sections are registered to be analyzed on the same plane, and average distances from all given CD8 cells to other immune cells or another CD8 cell were quantified. Each dot represents a separate tumor and adjacent normal region within the tissue from a total of 3-5 mice per arm. Results from one-way ANOVA and pair-wise testing shown as FDR-adjusted p values*<0.05, **<0.01, ***<0.001, ****<0.0001.

Supplementary Figure 7

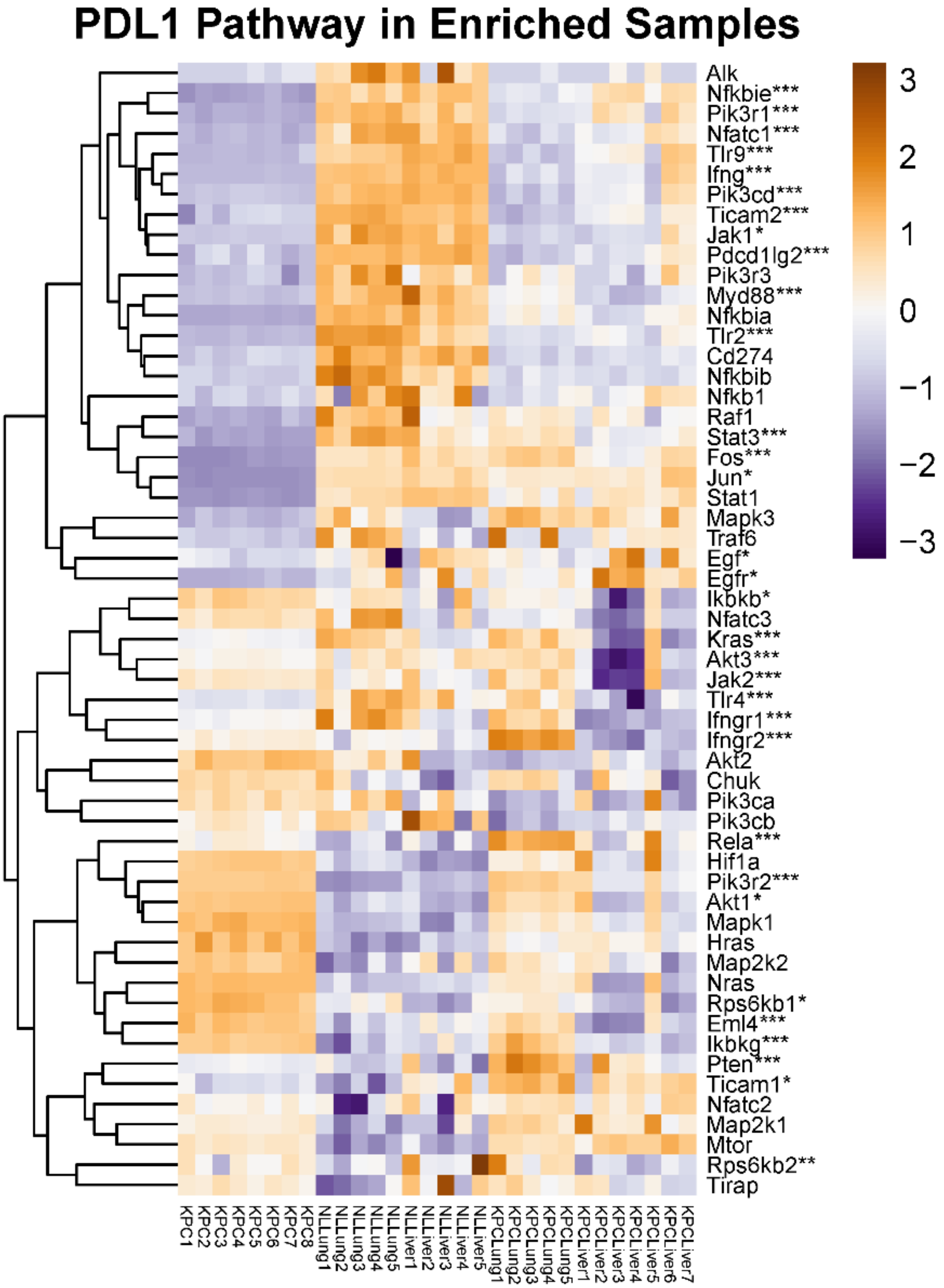

Heatmap of expression profiles for PDL1 pathway genes shown for every sample. All gene expression differential analyses were performed based on the negative binomial distribution using *DESeq2*. FDR adjusted P values *<0.05, **<0.01, ***<0.005 for comparison between the liver and lung TME sites are shown next to each gene name.

Supplementary Figure 8

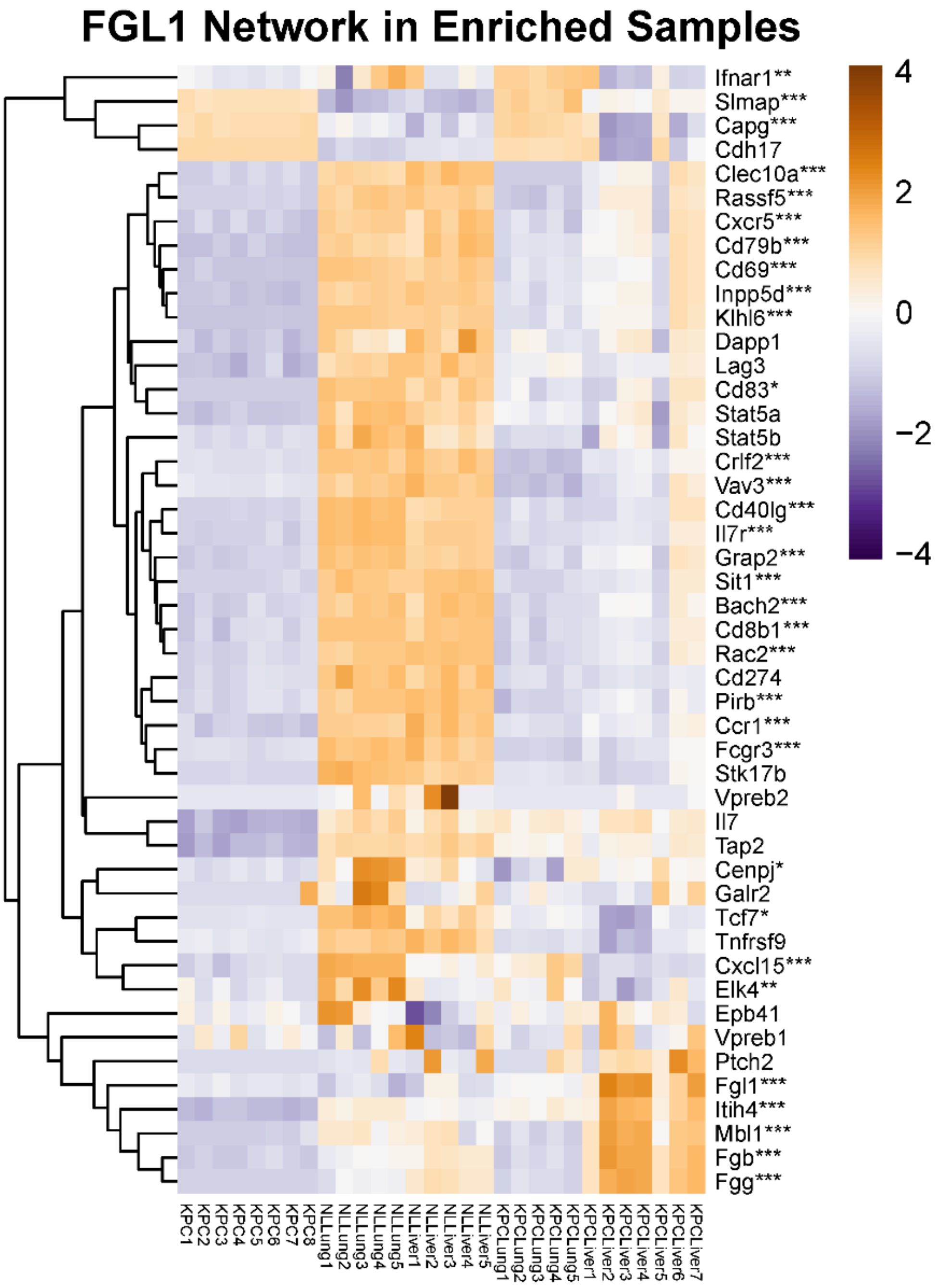

Heatmap of expression profiles for FGL1-Lag3 network genes shown for every sample. All gene expression differential analyses were performed based on the negative binomial distribution using *DESeq2*. FDR adjusted P values *<0.05, **<0.01, ***<0.005 for comparison between the liver and lung TME sites are shown next to each gene name.

Supplementary Figure 9

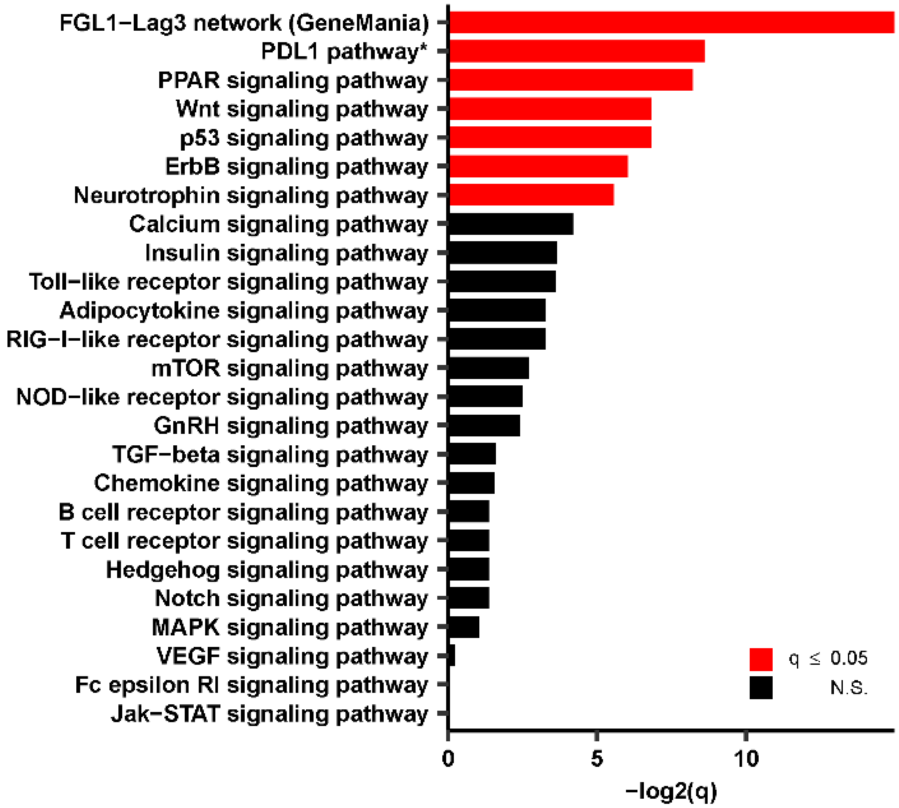

Results of pathway analysis. Enrichment for pathways in genes differentially expressed in liver and lung TMEs were tested based on mean-rank gene set enrichment. All pathways in the KEGG signaling pathway database along with KEGG PDL1 pathway and network of genes associated with FGL1-Lag3 based on GeneMania search were tested. Negative log2 of FDR adjusted P values (q values) are shown as barplots for all pathways, sorted from the highest to the lowest value. Significant q values (<0.05) are noted in red.

Supplementary Figure 10

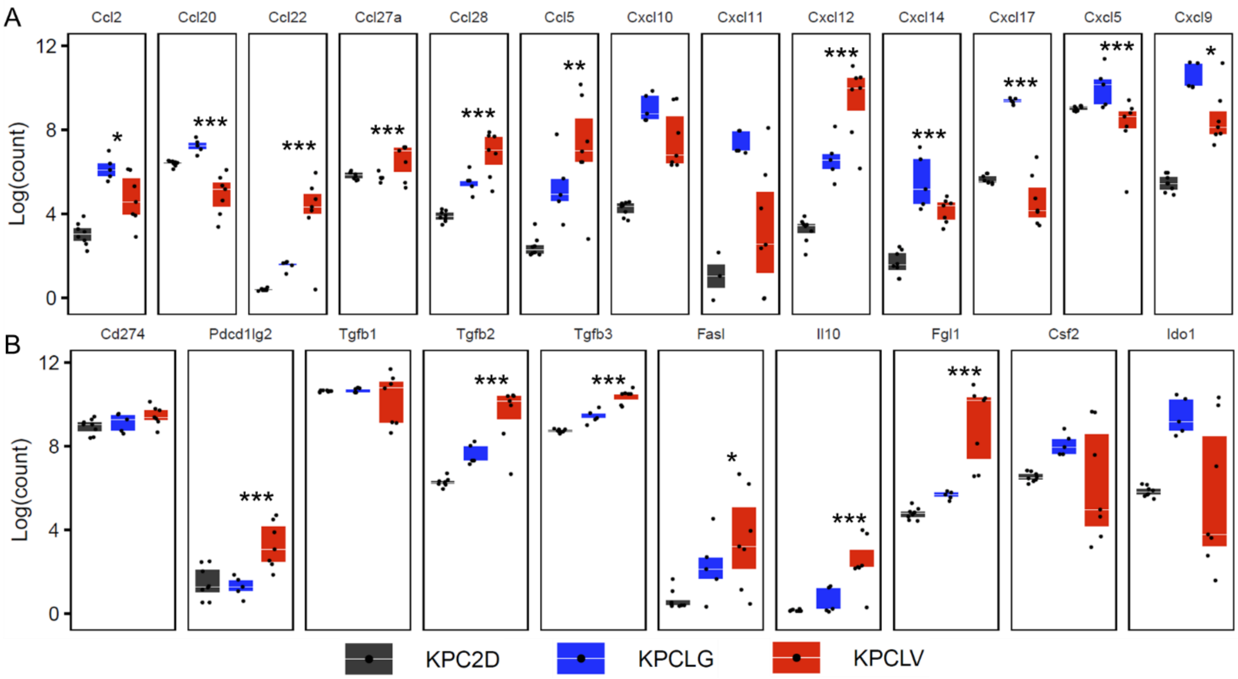

Expression of select genes from the non-immune compartment of the liver and lung TMEs. Boxplots for (A) chemokines and (B) immune-suppressor molecules are shown with median bars. Black boxes represent KPC cells from in vitro cultures, blue boxes represent KPC-bearing lung samples, and red boxes represent KPC-bearing liver samples. FDR-adjusted p values for comparison between the lung and liver TMEs by *DESeq2* are shown as *<0.05, **<0.01, ***<0.005.

Supplementary Figure 11

A

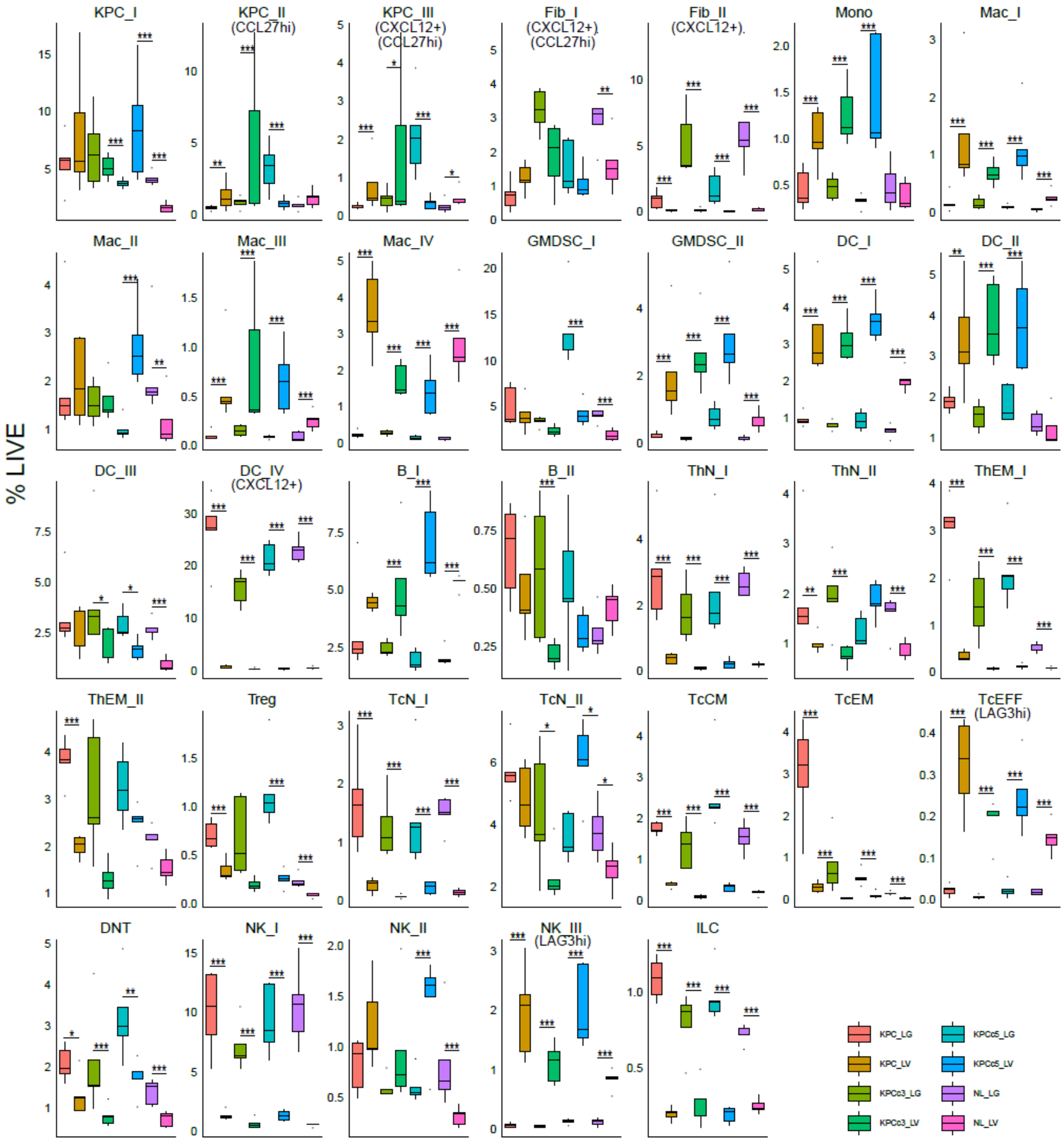

B

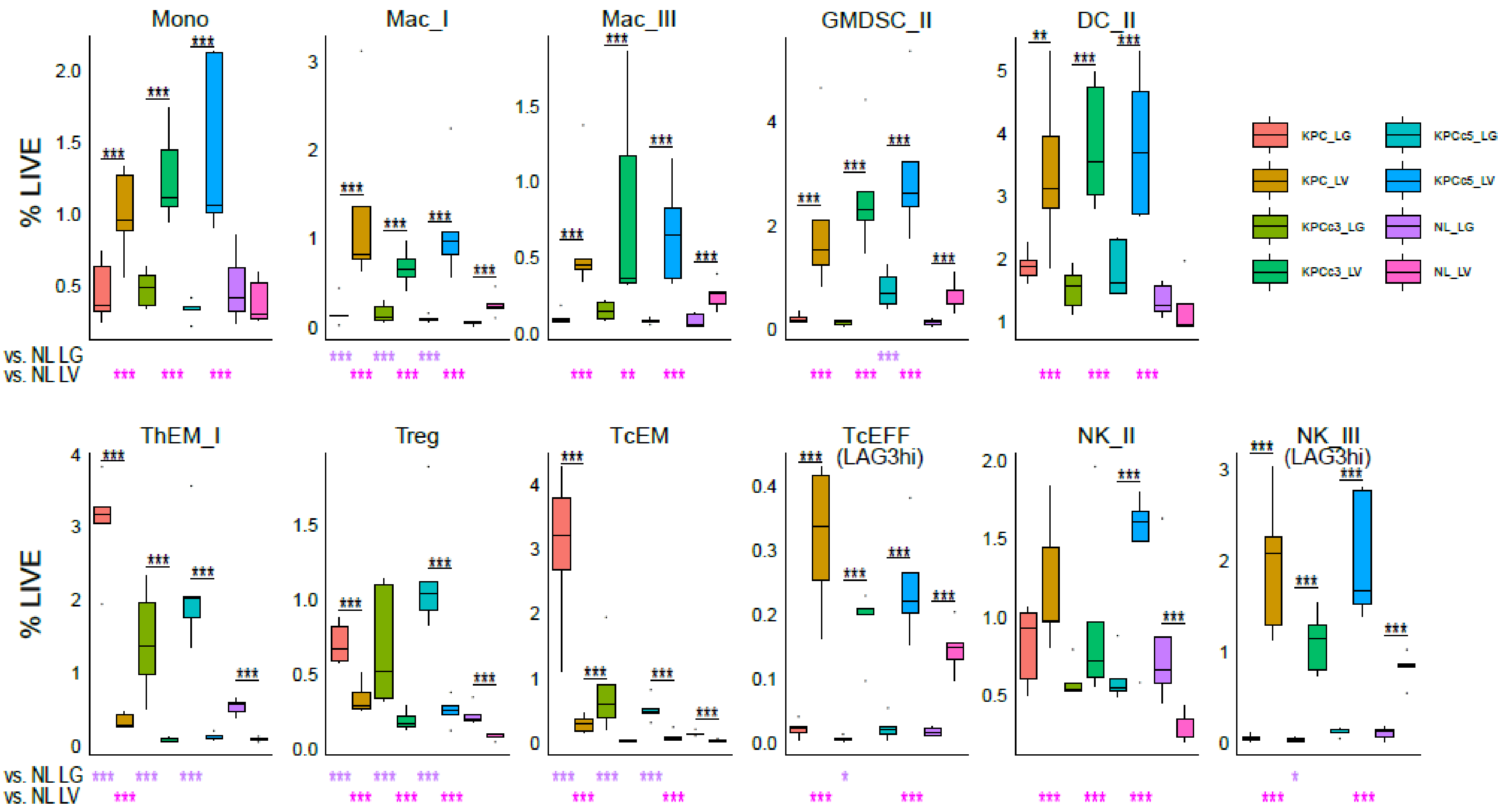
Mono
Mac_I
Mac_III
GMDSC_II
DC_II
ThEM_I
Treg
TcEM
TcEFF (LAG3hi)
NK_II
NK_III (LAG3hi)
% LIVE
vs. NL LG
vs. NL LV
KPC_LG
KPC_LV
KPCc3_LG
KPCc3_LV
KPCc5_LG
KPCc5_LV
NL_LG
NL_LV

C

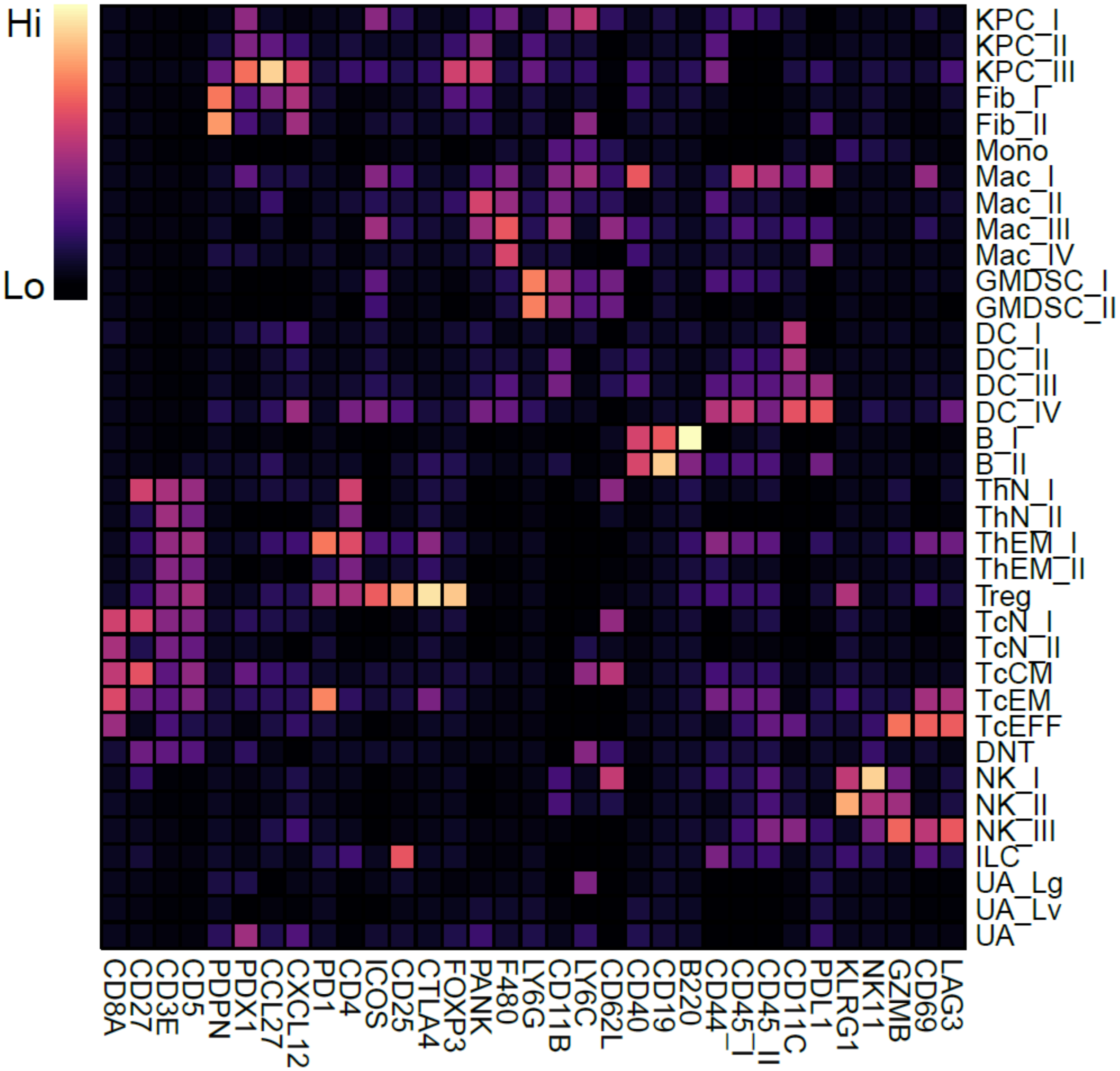

CyTOF profiling of three KPC models along with normal controls. (A-B) CyTOF analysis of KPC-bearing lung (KPC_LG), liver (KPC_LV), KPC 2838c3-bearing lung (KPCc3_LG) and liver (KPCc3_LV), KPC 6419c5-bearing lung (KPCc5_LG) and liver (KPCc5_LV), and normal lung (NL_LG) and liver (NL_LV) controls. Comparisons between tissue sites for abundances (% of total live cells) of all cell type clusters (A) along with comparisons to normal controls for select immune cell types (B) are shown. N=5 per group. Results from EdgeR shown as FDR-adjusted p values *<0.05, **<0.01, ***<0.005. (C) Expression profile heatmap for each cell type cluster. Markers used for the repeat CyTOF analysis using the three KPC models and normal tissue controls are shown as columns. Each row represents an annotated cell type cluster from the dataset. Expression levels are scaled by columns.

Supplementary Figure 12

A

Representative Human Liver Metastatic PDAC at 10X (scale bar: 100µm)

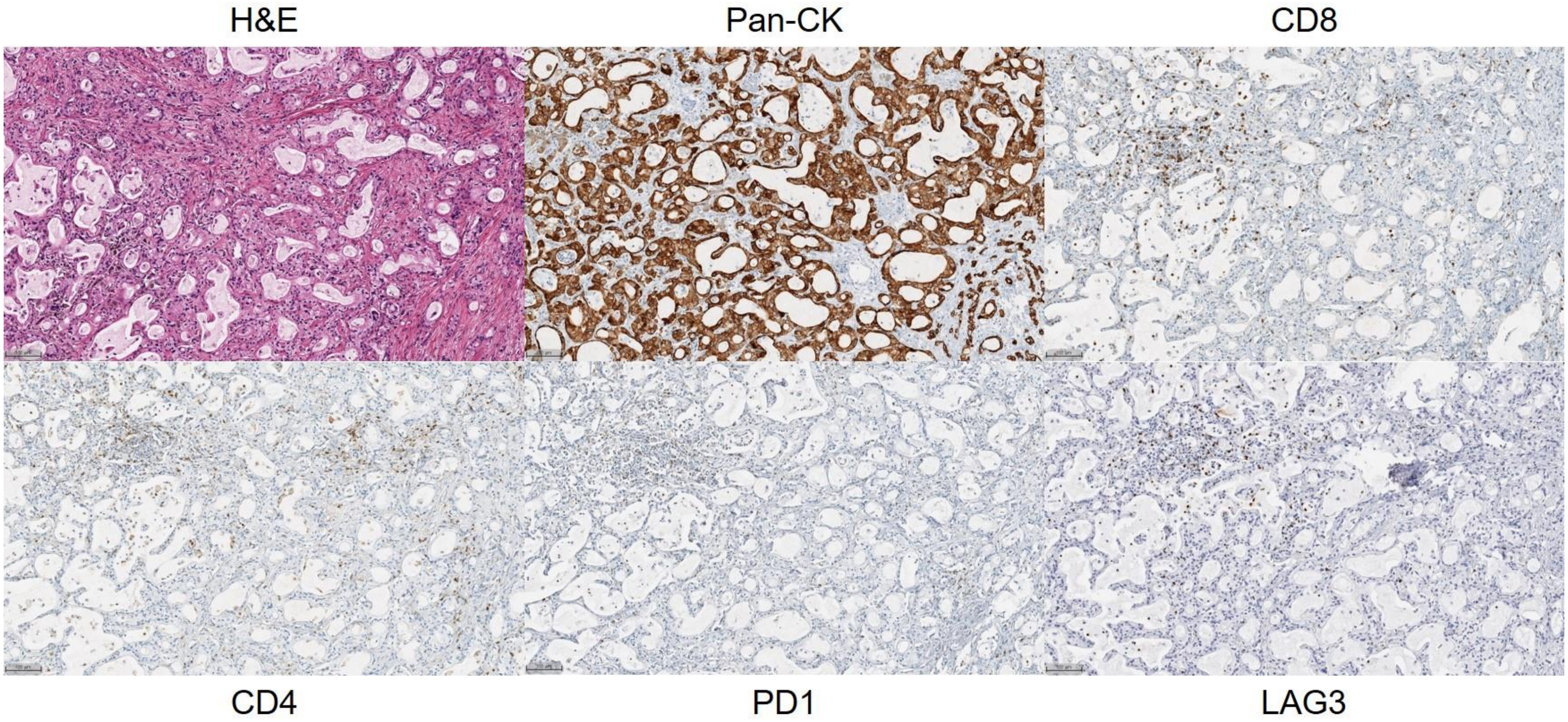

Representative Human Liver Metastatic PDAC at 20X (scale bar: 50µm)

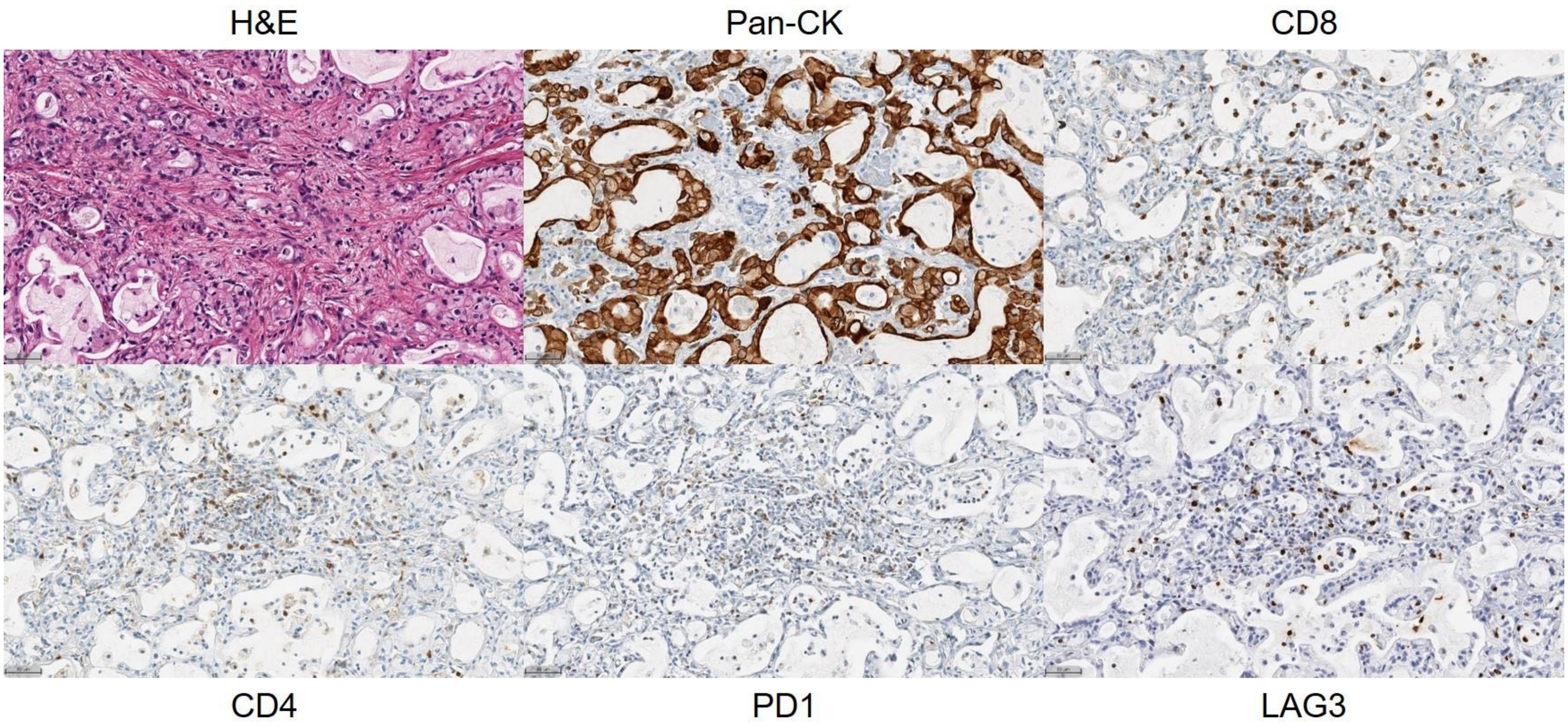

B

Representative Human Lung Metastatic PDAC at 10X (scale bar: 100µm)

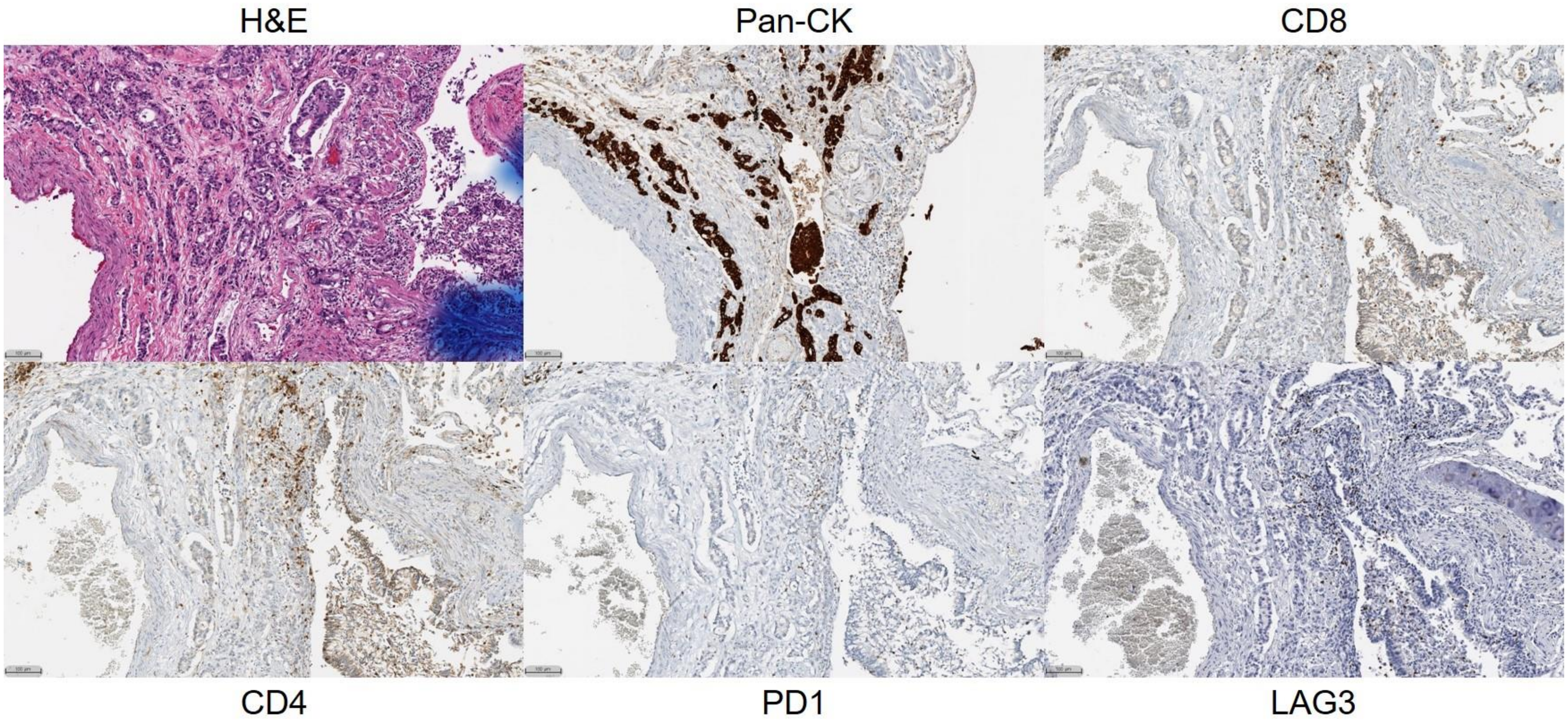

Representative Human Lung Metastatic PDAC at 20X (scale bar: 50µm)

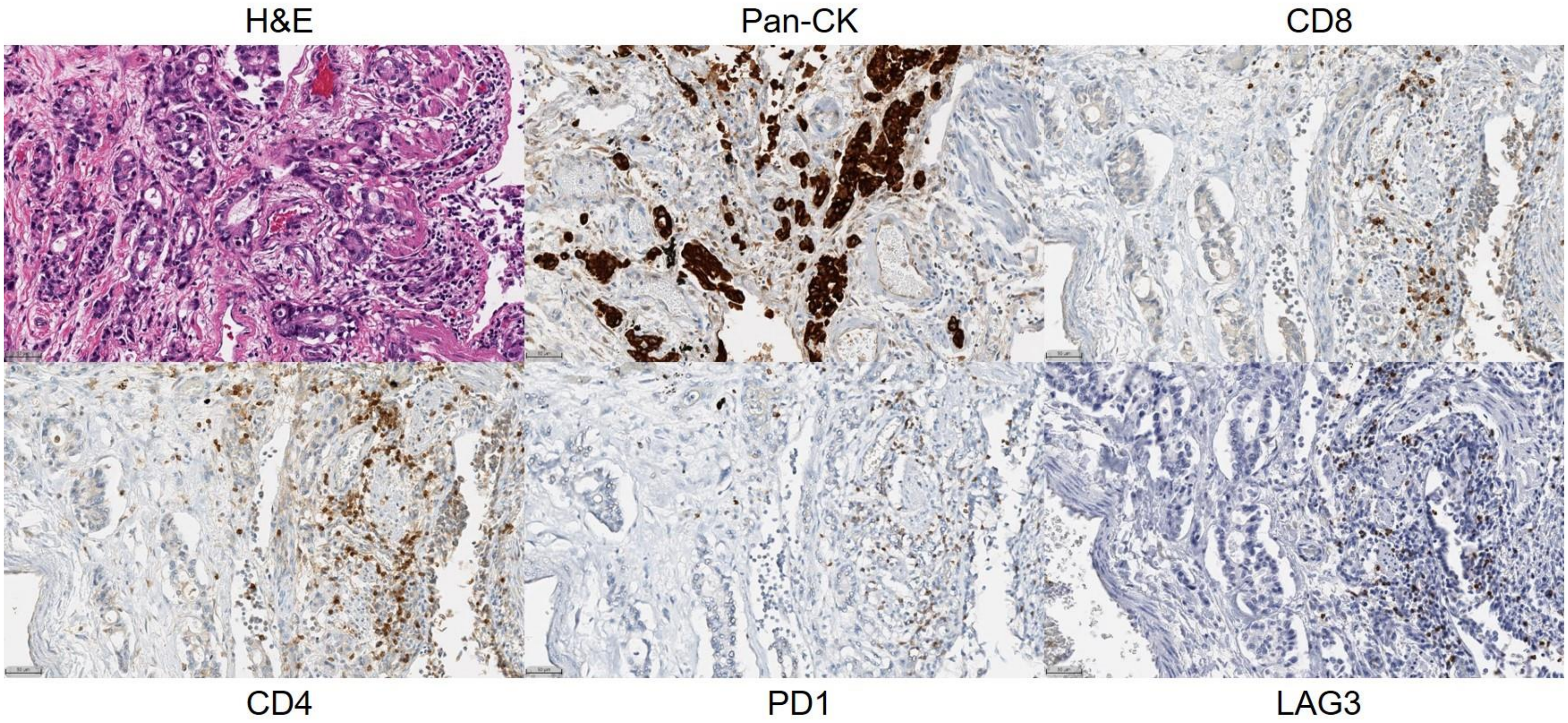

C

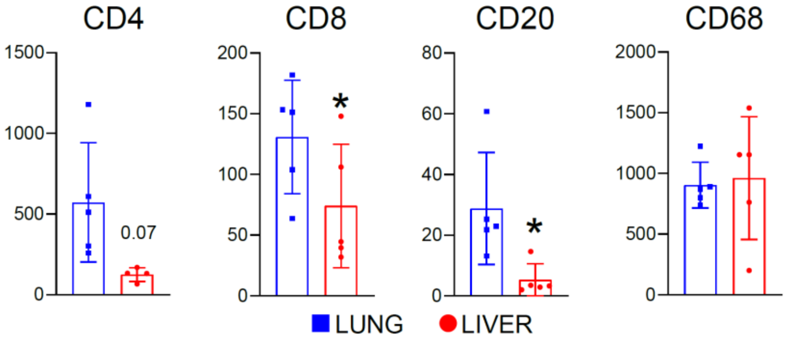
CD4
CD8
CD20
CD68
0.07
*
*
LUNG
LIVER

D

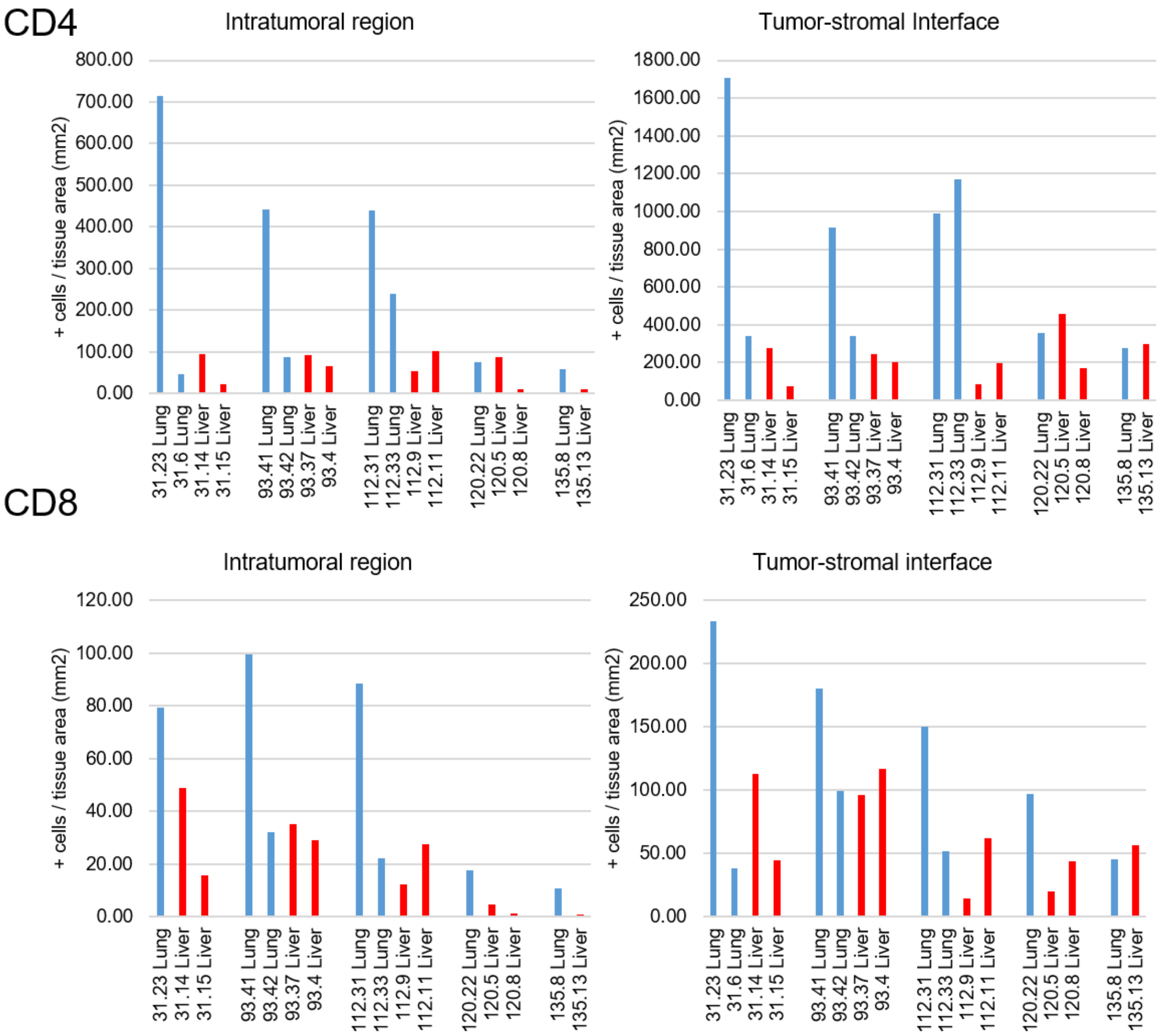
CD4
Intratumoral region
Tumor-stromal Interface
+ cells / tissue area (mm2)
31.23 Lung
31.6 Lung
31.14 Liver
31.15 Liver
93.41 Lung
93.42 Lung
93.37 Liver
93.4 Liver
112.31 Lung
112.33 Lung
112.9 Liver
112.11 Liver
120.22 Lung
120.5 Liver
120.8 Liver
135.8 Lung
135.13 Liver
CD8
Intratumoral region
Tumor-stromal interface
+ cells / tissue area (mm2)

E

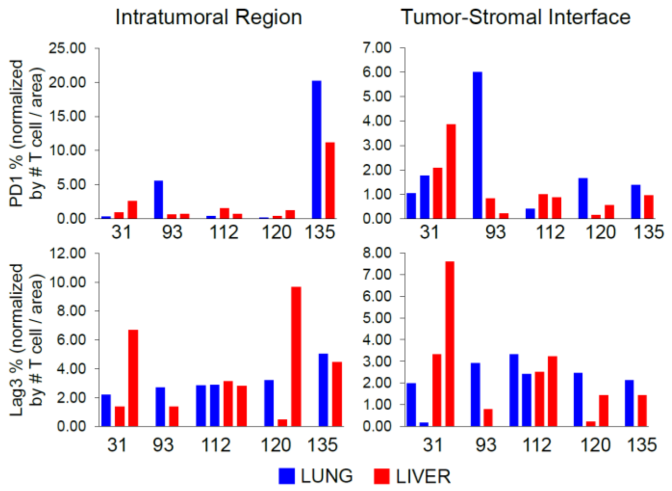

Representative pictures of patient-matched metastatic pancreatic ductal adenocarcinoma lesions in (A) liver and (B) lung from the Rapid Autopsy Database at Johns Hopkins for H&E staining and detection of cytokeratin, CD4, CD8, PD1, LAG3 by immunohistochemistry. Similar regions are shown at 10X (top) and 20X (bottom). (C) Immunohistochemical analysis of marker expression per mm2 of tumor area from five pairs of matched liver and lung metastatic PDAC samples are shown as open bar plots at means ± SEM with individual samples denoted by blue squares (lung) and red circles (liver). P values represent t-test paired by patients; *<0.05. (D) CD4 and CD8 expression in the TME by immunohistochemistry in individual samples illustrating inter-patient, inter-site, and intra-site variability. Positive expression per mm^2 of tissue area analyzed by HALO software are shown for both the intratumoral and tumor-stromal (i.e. peri-tumoral) regions. On the horizontal axis, numbers corresponding to each of the five patients (31, 93, 112, 120, 135) are annotated along with the numbers after the decimal point to indicate the specific sample number. (E) Positive expression of PD1 and Lag3 per the number of T cells per mm2 tissue area in the lung (blue) and liver (red) are shown on a per-sample basis. On the horizontal axis, numbers corresponding to each of the five patients (31, 93, 112, 120, 135) are annotated.

Supplementary Figure 13

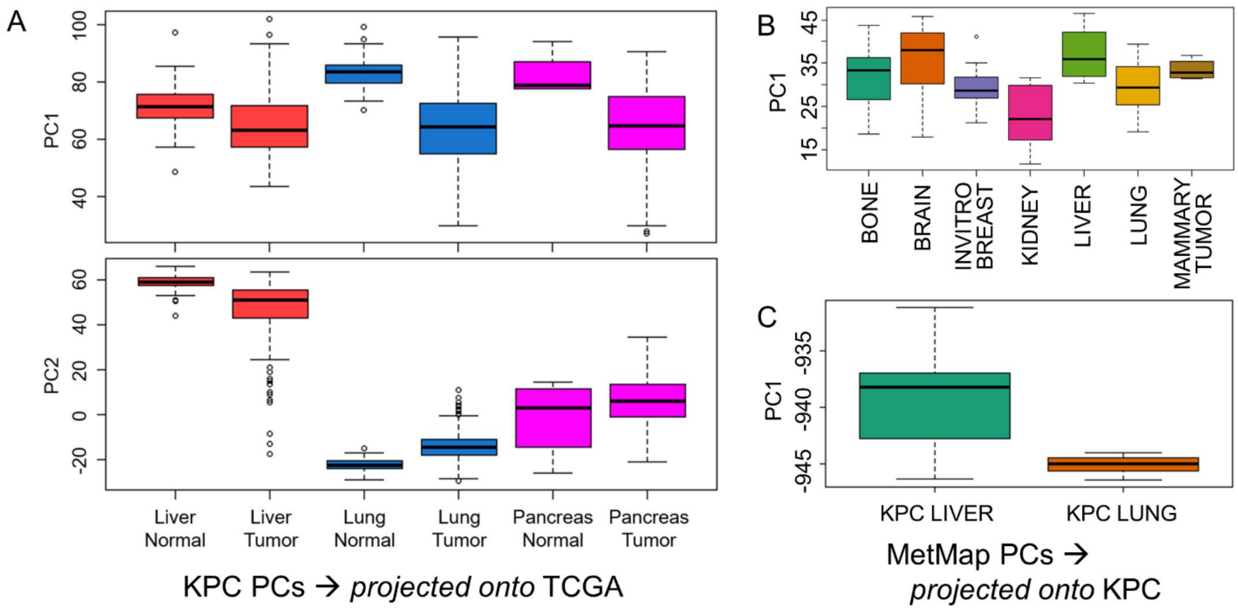

Cross-validation of site-specific signatures using disparate datasets. (A) Projection of principal component (PC) dimensions of the metastatic KPC mouse models (Figure 3B) to TCGA data. Transfer learning algorithm ProjectR was employed to project PC1 and PC2 axes derived from the metastatic KPC mouse models onto tumor and adjacent normal samples from the liver, lung, and pancreas TCGA datasets. (B) PC analysis of MetMap data showing metastatic cancer cells from liver most represented by PC1. (C) Projection of PC1 from MetMap onto KPC dataset recapitulates liver-dominant signal.
