# Supplementary material for: Multi-omic profiling of lung and liver tumor microenvironments of metastatic pancreatic cancer reveals site-specific immune regulatory pathways
Source: Genome Biol. 2021 May 13;22:154. doi: 10.1186/s13059-021-02363-6 (PMC8118107; doi:10.1186/s13059-021-02363-6)
Supplement: Supplementary file 8 — Additional file 8. Review history. [file 13059_2021_2363_MOESM8_ESM.docx]

Review History

**First round of review**

**Reviewer 1**

**Are you able to assess all statistics in the manuscript, including the appropriateness of statistical tests used?** No, I do not feel adequately qualified to assess the statistics.

**Comments to author:**

This is a very relevant study. The immune microenvironments of the lung and liver sites of pancreatic cancer metastasis are long suspected to impact on patient survival. The insights obtained bear high clinical relevance. The study uses unique materials and relevant models.

I do have some concerns that should be addressed:

Major concerns:

I understand that the focus of this study (and the journal to which it was submitted) is descriptive. Nevertheless, the study would gain so much from functional experiments in which the candidate chemokines identified are tested. Given that the authors have access to the KPC cells and can graft these with great success, I would think that a gene editing approach prior to another grafting experiment is not a very tall order. In lieu of such data, the paper remains descriptive.

To be truly informative, the above should include a panel or library of gRNAs (or hairpins) to provide a semi-unbiased confirmation of the chemokines that govern the observed differences between the metastatic sites.

The difference in immune composition between healthy organs and the matching metastasis tissue (e.g. Figure 1D) is very modest. Is this truly expected? Is the impact of having tumor cells in those tissues really that minimal? I suspect that the KPC cells are insufficiently immunogenic to elicit a real response. Is there any way the authors can address this, perhaps by using a different mouse strain or other cells to be grafted.

Figure 4; which cells express these candidate chemokines? This is very descriptive, would be good to get a little more depth here.

Minor comments:

There are a lot of Supplemental figures. I think this could be made more concise for instance by merging conceptually connected panels that are now in different figures. In all earnest, I do not think the KEGG and Cytoscape analyses in S7 and S9 are very valuable.

Callout for Figure 1E is missing?

Figure 1B; any way to benchmark/verify these profiles by measuring isolated B cells, Tregs, etc. ?

**Reviewer 2**

**Are you able to assess all statistics in the manuscript, including the appropriateness of statistical tests used?** No, I do not feel adequately qualified to assess the statistics.

**Comments to author:**

In this manuscript, the authors investigate molecular and cellular components unique to the microenvironment in the lung and liver in the setting of pancreatic cancer (PDAC). Their goal was to compare immune suppressive pathways operative in these distinct anatomic regions (i.e. lung and liver). To do this, they used a range of samples including mouse PDAC tumors, paired human lung and liver metastatic PDAC samples, rapid autopsy PDAC specimens of lung and liver mets, and the TCGA dataset. From their studies, they conclude that "distinctive immune-suppressive features" define the liver and lung tumor microenvironments of PDAC. Overall, the study sets out to address an important question regarding the significance of anatomic location on the immune response to metastatic PDAC. However, multiple technical limitations in the study design and analysis as well as limitations in the interpretation reduce the significance of the findings.

1. In Figure 1, the authors use an animal model with one cell line to study the immune microenvironment of liver and lung metastases. They first show that the immune microenvironment of the normal liver and lung is remarkably distinct. They then compare these organs in the presence of PDAC metastasis and show changes in the immune compartments. They ultimately conclude on line 10 of page 6 that the lung tumor microenvironment (TME) is composed of a higher level of immune cell infiltration and activation than the liver TME. There are several technical limitations to this conclusion including (i) it is expected that the normal liver and lung environments will be altered in the setting of cancer - numerous studies have published on this as a pre-metastatic niche formation. As a result, the changes observed cannot be concluded to be specific to the TME as opposed to the normal tissue microenvironment, (ii) the CYTOF analyses do not permit spatial analyses to understand where the observed immune changes are occurring (i.e. in the normal tissue parenchyma or in the metastatic TME), and (iii) only one cell line is studied - it is unclear whether the results are generalizable to other cell lines. Notably, KPC cell lines can differ significantly in the microenvironment that they form. The name of the cell line used is also not indicated. Finally, the authors could have considered comparing the lung and liver microenvironments in the KPC autochthonous model (with comparison to normal PC or C littermates). This would allow for understanding of how much of the changes observed in their model reflect tumor cells and the TME versus changes in the normal liver/lung microenvironments as a result of cancer development in the pancreas.

2. After demonstrating in Figure 1 that the normal lung and liver immune microenvironments differ dramatically, the authors no longer consider this biology in their subsequent analyses which is problematic given that differences between lung and liver specimens include normal parenchymal tissue. For example, in Fig 2, the authors show that Lag3+ CD8 T and NK cells are more abundant in the liver compared to lung, but this may reflect the differences in the normal lung and liver microenvironments. In general, findings observed may not be related to the TME but rather the organ parenchyma. Validation of genes and their expression spatially within tumor and parenchyma is not presented. The tumor-antigen specificity of these T cells is also unclear and so the relevance to the tumor is uncertain.

3. In Figure 3, the authors then perform RNAseq on multiple tissue specimens from their mouse model of liver and lung metastasis. While the samples studied lack immune cells due to flow sorting, the samples do contain normal parenchymal cells such as hepatocytes and pneumocytes in liver and lung, respectively. As a result, multiple cell types are included in addition to tumor cells. It is not clear how the authors normalize for the presence of the different cell populations present and the fact that the liver and lung normal parenchyma will change in the presence of tumor and the tumor cells will also adapt to the microenvironment. Validation of the genes identified is not provided with exception of Lag3 (by Cytof and Flow) which the authors identify as significantly different between lung and liver tissue (spatial orientation of Lag3 is unclear though). They also show that Fgl1 is increased but it is unclear which cells are expressing this molecule.

4. There is no statistical section to the manuscript.

5. The authors also study the immune microenvironment of human PDAC from rapid autopsy specimens/database and TCGA datasets. The IHC shown in Fig S11 would benefit from a cytokeratin stain to demonstrate where the tumor cells are. The images are not of high enough resolution to permit interpretation of the H&Es or IHC stains. The sample size to make conclusions on the intratumoral density of immune infiltrates in distinct lesions is small and differences are modest especially in light of the heterogeneity portrayed in Fig S12.

6. Images in Fig S13B and S13C are the same except PD-1 in B and Lag3 in C. It would be easier to interpret if the images were not duplicated and Lag3 image was included as part of B. The resolution of the images is insufficient to allow for interpretation. Hi-mag insets would help too. It also appears that the differences in CD8 and subsequent Lag3 may represent focal accumulation of CD8 T cells rather than diffuse infiltration. Does this match with histology in the mouse model? Is Lag3 also increased in the normal parenchymal regions adjacent to tumor lesions?

7. For the RNAseq from autopsy database, many of the immune cell coefficients analyzed were undetectable in samples lending to few interpretable specimens. How was the quality of the RNA analyzed considered - for example, presence of CD45 transcripts of CK19 transcripts? Its also not clear how the authors conclude from this dataset that "these findings from human sample datasets largely corroborate higher T cell density in the lung TME and the immune-suppressed state within the liver TME".

**Reviewer 3**

**Are you able to assess all statistics in the manuscript, including the appropriateness of statistical tests used?** Yes, and I have assessed the statistics in my report.

**Comments to author:**

General comments

The manuscript by Ho and colleagues describes a study using CyTOF and RNA-seq data to analyze lung and tumor microenvironments in metastatic pancreatic cancer. The authors first used a mouse model and CyTOF to characterize the infiltrating immune cells in lung and liver metastasis and then RNA-seq to study the non-immune compartment. The TME in the lung was composed of a higher level of immune cell infiltration and activation, whereas the non-immune compartment of the liver TME was relatively immune-suppressed compared to the lung. Human samples from an autopsies were analyzed using IHC and another published RNA-seq data was analyzed using deconvolution algorithm and some of the findings were corroborated. Finally, TCGA data was analyzed using transfer algorithm and deconvolution.

Albeit the study is of potential interest and might have translational implications, there are several concerns with the methodology as well as with the data analysis as described below. Specifically, the impact of the model on the TME needs to be analyzed in depth. It is necessary to provide the complete mutational profile of the used cell line (e.g. using exome-seq) since it is well known that the 1) mutational load/neoantigen load is associated with increased immune cell infiltration, and 2) a number of cancer driver genes are associated with the immune contexture (see PMID: 29562192). This information is of utmost importance for the interpretation of the results. Another concern is the use of a deconvolution method MIXTURE which is not yet published and apparently validated using only simulated and benchmark data sets (according to the bioRxiv manuscript). There are several recently published deconvolution methods that enable robust analysis (see review PMID: 31515541). Hence, the authors should reanalyze the data with a published method of choice. Finally, several specific comments need to be addressed.

Specific comments

1. Several papers analyzed RNA-seq data from the TCGA with respect to the immune cell infiltration (e.g. PMID: 31433971). Are the results of the current study (page 10) confirming previous findings?

2. The ErbB finding (page 7, line 20) should be either discussed in more detail or omitted.

3. The interplay between the immune and non-immune compartments of the TME is intriguing. The authors should consider using the information from a ligand-receptor database CellPhone.DB (PMID: 32103204) in order to provide additional evidence.

4. One innovative aspect is the use of the transfer learning approach. In the original publication mouse scRNA seq data from developing retina was used. The use of the method in a different domain (mouse-human immune cells) needs thorough discussion**.**

**Authors Response**

**Point-by-point responses to the reviewers’ comments:**

***Reviewer #1:*** *This is a very relevant study. The immune microenvironments of the lung and liver sites of pancreatic cancer metastasis are long suspected to impact on patient survival. The insights obtained bear high clinical relevance. The study uses unique materials and relevant models. I do have some concerns that should be addressed:*

*Major concerns:*

1. *I understand that the focus of this study (and the journal to which it was submitted) is descriptive. Nevertheless, the study would gain so much from functional experiments in which the candidate chemokines identified are tested. Given that the authors have access to the KPC cells and can graft these with great success, I would think that a gene editing approach prior to another grafting experiment is not a very tall order. In lieu of such data, the paper remains descriptive. To be truly informative, the above should include a panel or library of gRNAs (or hairpins) to provide a semi-unbiased confirmation of the chemokines that govern the observed differences between the metastatic sites.*

We agree with the reviewer’s comments that this study is descriptive in essence. We have generated and explored a combination of parallel and orthogonal datasets to make robust observations. We posit that these observations provide important insights in cancer immunology. While we agree that providing functional validation of the observed targets would significantly expand the conclusions we can make, we also believe that performing the studies in vivo in the approach suggested by the reviewer, would further warrant additional experiments for the rigor and robustness necessary to fully test the many hypothesized biological pathways. We submit that this is beyond the scope of the current study. However, we refer to the following key studies that have demonstrated that targeting CXCR4/CXCL12 (Feig et al., PNAS 2013, PMID 24277834 and Chen et al., Hepatology 2015 PMID 25529917), which is noted to be immunosuppressive features within the liver tumor microenvironment in the current study, enhances antitumor immune responses against models of pancreatic and liver cancers. These independent studies at least provide some functional validity of these observed targets. We have added these comments and references to the discussion section in the final paragraph.

2. *The difference in immune composition between healthy organs and the matching metastasis tissue (e.g. Figure 1D) is very modest. Is this truly expected? Is the impact of having tumor cells in those tissues really that minimal? I suspect that the KPC cells are insufficiently immunogenic to elicit a real response. Is there any way the authors can address this, perhaps by using a different mouse strain or other cells to be grafted.*

The reviewer correctly points out the limited immunogenicity of KPC cells. This is further complicated by the limitations of the assay relying on enzymatic dissociation of the entire liver or lung tissue, thereby including all of the surrounding normal tissues within the sample. A pertinent question of robustness of the findings associated with using one cell line is also raised. To address these comments, we have performed two additional investigations: (1) we have performed immunohistochemistry (IHC) of the KPC tumor-bearing liver and lung samples to characterize the immune cell densities intratumorally and also in normal adjacent regions; (2) we have repeated the CyTOF immune profiling of the KPC tumors but also along with two other KPC tumor models to enhance the rigor of the findings. IHC analysis revealed that the density of immune cells are significantly higher intratumorally, especially in the KPC-bearing lungs. When incorporating two additional KPC cell lines (2838c3 or “c3” and 6419c5 or “c5”; Li et al., Immunity 2018, PMID 29958801), we have found that the immune profiles recapitulate our original observations reported in the manuscript; many immunosuppressive cell populations as well as antigen-experienced populations are increased in the tumor-bearing conditions compared to the normal controls, and T cells are more richly infiltrating the tumor microenvironment in the lungs compared to the lung, whereas LAG3-high T and NK cell subpopulations are greater in the liver. Both of these results are summarized below and now also added to the revised manuscript as Supplementary Figures 6, 11.

**CyTOF immune profiling of three KPC models along with normal controls.** CyTOF analysis of KPC-bearing lung (KPC_LG), liver (KPC_LV), KPCc3-bearing lung (KPCc3_LG) and liver (KPCc3_LV), KPCc5-bearing lung (KPCc5_LG) and liver (KPCc5_LV), and normal lung (NL_LG) and liver (NL_LV) controls. N=5 per group. Results from EdgeR shown as FDR-adjusted p values *<0.05, **<0.01, ***<0.005.

**Density of immune cells in adjacent normal regions and within tumors in the metastatic KPC mouse models.** Immunohistochemistry (IHC) of CD8, CD4, CD68, and B220 are performed and quantified as number of positive cells per tissue area (mm^2^) in the KPC tumors within lungs (KPC LG) and livers (KPC LV) and the normal adjacent regions in the lungs (NL LG) and livers (NL LV). Each dot represents a separate tumor and adjacent normal region within the tissue from a total of 3-5 mice per arm. Results from one-way ANOVA and pair-wise testing shown as FDR-adjusted p values*<0.05, **<0.01, ***<0.001, ****<0.0001.

3. *Figure 4; which cells express these candidate chemokines? This is very descriptive, would be good to get a little more depth here.*

RNAseq analysis demonstrated that some cytokines were particularly predominant in the KPC-bearing liver tumor microenvironments including CCL27, CCL28, and CXCL12. In the same CyTOF experiment shown above in which we repeated the immune profiling using three different KPC models, we have added CCL27- and CXCL12-specific antibodies into the panel to better understand their cellular sources as the reviewer suggested, shown in the Figure below. We have found that they are primarily arising from the non-immune compartment, highly expressed by a KPC cell cluster, “KPC_III”. This adds support to the importance of the KPC behavior shaped by the tumor microenvironment. This figure has also been incorporated into the revised manuscript as a panel in Supplementary Figure 11C.

**CyTOF expression profile heatmap for each cell type cluster**. Markers used for the repeat CyTOF analysis using the three KPC models and normal tissue controls are shown as columns. Each row represents an annotated cell type cluster from the dataset. Expression levels are scaled by columns.

*Minor comments:*

1. *There are a lot of Supplemental figures. I think this could be made more concise for instance by merging conceptually connected panels that are now in different figures. In all earnest, I do not think the KEGG and Cytoscape analyses in S7 and S9 are very valuable.*

We appreciate this suggestion have removed these figures to streamline the presentation of data as suggested.

2. *Callout for Figure 1E is missing?*

We have revised the manuscript to correct the missing callout.

3. *Figure 1B; any way to benchmark/verify these profiles by measuring isolated B cells, Tregs, etc. ?*

The set of antibodies used for the profile shown on Figure 1B are well-established clones, and the canonical markers used for distinguishing the cell types are also robustly documented in the immunology literature (e.g. Gubin et al., Cell 2018, PMID 30343900); each cell type is annotated based on canonical expression profiles, e.g. CD3^+^CD4^+^ is annotated as helper T cells. Based on the reviewers’ comment, we have also visualized the expression of equivalent genes from publicly available RNAseq database compiled from flow sorted cells (ImmGen) that are similar to the cell types identified in this CyTOF dataset. This heatmap is consistent with our CyTOF dataset. While we do not believe that this type of figure would add significantly to the results of the study, we have added an additional explanation of what we mean by “annotating” these clusters into the results section under the subheading *Immune profiling with mass cytometry reveals enhanced immune activation in lung metastases*.

**Benchmarking of the CyTOF dataset.** Gene expression profiles from ImmGen database for the CyTOF marker set used to determine cell types. Cells are sorted by flow cytometry prior to performing RNAseq: B (B_Sp), helper T (T_4_Th), naïve helper T (T_4_Nve_Sp), cytotoxic T (T_8_Th), naïve cytotoxic T (T_8_Nve_Sp); central memory (T8_Tcm_LCMV) and effector memory (T8_Tem_LCMV) cytotoxic T cells in response to lymphocytic choriomeningitis virus, regulatory T (Treg_4_FP3+), NK (NK_27-11b+_Sp), dendritic cells (DC_4+_Sp, DC_8+_Sp), macrophages (MF_RP_Sp), monocytes (Mo_6C+II-_Bl), granulocytes (GN_Sp. Genes shown: *Cd3e* (CD3), *Cd8a* (CD8), *Cd19* (CD19), *Itgax* (CD11c), *Cd4* (CD4), *Itgam* (CD11b), *Ly6g* (Ly6G), *Foxp3* (Foxp3), *Rorc* (Rorgt), *Ly6c1* (Ly6C), *Sell* (CD62L), *Cd44* (CD44), *Klrb1* (CD161; NK1.1). Diverging color heatmap is based on scaled expression with: Red (high) – Yellow (mid) – Blue (low).

***Reviewer #2:*** *In this manuscript, the authors investigate molecular and cellular components unique to the microenvironment in the lung and liver in the setting of pancreatic cancer (PDAC). Their goal was to compare immune suppressive pathways operative in these distinct anatomic regions (i.e. lung and liver). To do this, they used a range of samples including mouse PDAC tumors, paired human lung and liver metastatic PDAC samples, rapid autopsy PDAC specimens of lung and liver mets, and the TCGA dataset. From their studies, they conclude that "distinctive immune-suppressive features" define the liver and lung tumor microenvironments of PDAC. Overall, the study sets out to address an important question regarding the significance of anatomic location on the immune response to metastatic PDAC. However, multiple technical limitations in the study design and analysis as well as limitations in the interpretation reduce the significance of the findings.*

1. *In Figure 1, the authors use an animal model with one cell line to study the immune microenvironment of liver and lung metastases. They first show that the immune microenvironment of the normal liver and lung is remarkably distinct. They then compare these organs in the presence of PDAC metastasis and show changes in the immune compartments. They ultimately conclude on line 10 of page 6 that the lung tumor microenvironment (TME) is composed of a higher level of immune cell infiltration and activation than the liver TME. There are several technical limitations to this conclusion including (i) it is expected that the normal liver and lung environments will be altered in the setting of cancer - numerous studies have published on this as a pre-metastatic niche formation. As a result, the changes observed cannot be concluded to be specific to the TME as opposed to the normal tissue microenvironment, (ii) the CYTOF analyses do not permit spatial analyses to understand where the observed immune changes are occurring (i.e. in the normal tissue parenchyma or in the metastatic TME), and (iii) only one cell line is studied - it is unclear whether the results are generalizable to other cell lines. Notably, KPC cell lines can differ significantly in the microenvironment that they form. The name of the cell line used is also not indicated. Finally, the authors could have considered comparing the lung and liver microenvironments in the KPC autochthonous model (with comparison to normal PC or C littermates). This would allow for understanding of how much of the changes observed in their model reflect tumor cells and the TME versus changes in the normal liver/lung microenvironments as a result of cancer development in the pancreas.*

We thank the reviewer for these important points. To address points (i) and (ii) which are related to the limitation of our data providing insights directly related to the tumor microenvironment and the spatial arrangement of cells within the tumor, we have added an additional paragraph within the Results subsection *Immune profiling with mass cytometry reveals enhanced immune activation in lung metastases* to describe our new analysis of the KPC-bearing lungs and livers using immunohistochemistry to analyze the differences in immune cell densities within the tumor directly and also in the nearby normal tissue. We have also in addition used these data to analyze the distance from a given CD8 T cell to a CD4+ T cell, CD68+ myeloid cell, B220+ B cell, and another CD8+ T cell to demonstrate that the spatial proximities of these immune cells are also altered. All of these analyses are now shown on Supplemental Figure 6. Furthermore, we have added a more in-depth discussion on the value of emerging, more high-dimensional spatial technologies in understanding the TME within the second paragraph of the discussion section.

**Proximity of immune cells in adjacent normal regions and within tumors in the metastatic KPC mouse models.** Immunohistochemistry (IHC) of CD8, CD4, CD68, and B220 are performed. Using HALO, adjacent sections are registered to be analyzed on the same plane. Average distance from all given CD8 cells to other immune cells or another CD8 cell quantified within each adjacent normal or intratumoral regions are calculated (µm) and averaged per region. All average distances are compared among the regions from the KPC tumors within lungs (KPC LG) and livers (KPC LV) and the normal adjacent regions in the lungs (NL LG) and livers (NL LV). Each dot represents a separate tumor and adjacent normal region within the tissue from a total of 3-5 mice per arm. Results from one-way ANOVA and pair-wise testing shown as FDR-adjusted p values*<0.05, **<0.01, ***<0.001, ****<0.0001.

To address point (iii) regarding the single cell line study, we have performed additional profiling on liver and lung metastatic sites established using two other KPC cell lines as shown also above in response to Reviewer 1’s major comment #2, which is now incorporated into the manuscript as Supplementary Figure 11. Finally, with regards to the comment about using an autochthonous model, we agree that this would be particularly interesting and biologically a very important experiment. However, the comparisons within the autochthonous model would not enable rigorous understanding of the site-specific differences, which is what we wanted to learn in the current study, since: (i), the timing of these metastatic events cannot be controlled, and (ii), the successful metastatic lesions would be clonally heterogeneous and thus the immune responses may be diverse. Thus, we also describe these challenges in the second to last paragraph of the discussion section of the revised manuscript to highlight that while the autochthonous model is highly clinically relevant, there would be technical caveats to the model which would make the data interpretation particularly challenging.

2. *After demonstrating in Figure 1 that the normal lung and liver immune microenvironments differ dramatically, the authors no longer consider this biology in their subsequent analyses which is problematic given that differences between lung and liver specimens include normal parenchymal tissue. For example, in Fig 2, the authors show that Lag3+ CD8 T and NK cells are more abundant in the liver compared to lung, but this may reflect the differences in the normal lung and liver microenvironments. In general, findings observed may not be related to the TME but rather the organ parenchyma. Validation of genes and their expression spatially within tumor and parenchyma is not presented. The tumor-antigen specificity of these T cells is also unclear and so the relevance to the tumor is uncertain.*

**TCR repertoire analysis in normal livers and lungs and KPC-bearing livers and lungs.** T cell clonality for each sample is shown. N=3-6 per group. Results of unpaired t-tests shown. P-value *<0.05, ***<0.005.

We agree with the reviewer that it is important to show the normal controls in Figure 2 as well. In T cell subtype analysis, we have added the normal controls and now demonstrate the changes that occur due to the tumor which include: decreased naïve cytotoxic T cells in both metastatic sites; increased PD1+ effector memory populations in both metastatic sites; increased Treg populations in both metastatic sites; and Lag3+ effector T cell population in the liver only. We further support the tumor-driven nature of many of the immune cell differences as well as the spatial information of these immune cells by immunohistochemistry experiments as shown above. This updated figure is now shown in Figure 2. To further address the question about the tumor-antigen specificity of the T cells, we have profiled the T cell repertoires within normal livers and lungs as well as KPC-bearing livers and lungs. We found that the clonality of T cells within the tissue was significantly increased with the presence of KPC tumors, which is consistent with the observation that there are decreases in naïve and increases in memory T cells, i.e. more T cells have experienced antigens related to the tumor. This T cell repertoire data is now included as part of Figure 2 in the Revised manuscript.

3. *In Figure 3, the authors then perform RNAseq on multiple tissue specimens from their mouse model of liver and lung metastasis. While the samples studied lack immune cells due to flow sorting, the samples do contain normal parenchymal cells such as hepatocytes and pneumocytes in liver and lung, respectively. As a result, multiple cell types are included in addition to tumor cells. It is not clear how the authors normalize for the presence of the different cell populations present and the fact that the liver and lung normal parenchyma will change in the presence of tumor and the tumor cells will also adapt to the microenvironment. Validation of the genes identified is not provided with exception of Lag3 (by Cytof and Flow) which the authors identify as significantly different between lung and liver tissue (spatial orientation of Lag3 is unclear though). They also show that Fgl1 is increased but it is unclear which cells are expressing this molecule.*

We have developed a combined experimental and computational strategy to address the astute points raised by the reviewer as follows:

(1) We have recognized this and directly had shown in our original manuscript (and remains the same in the revised manuscript) on Figure 3 panel C that the expression of KPC-related genes (*Muc1*, *Cdh1*, *Krt18*, *Muc5ac*, *Krt19*, *Pdx1*) are enriched in the tumor-bearing tissue samples and that the tissue-specific genes (*Sftpa1*, *Sftpb* for lung; *F2*, *Fgb* for liver) are also very well represented in these samples.
(2) To further delineate the site-specific signals that arise from the tumor cells – but not parenchymal or stromal cells – within the KPC RNAseq data, we applied our transfer learning approach to compare our data with MetMap. This is a publicly available dataset based on RNA-seq profiling of uniquely barcoded breast cancer cell lines injected in vivo for spread onto five different metastatic sites. Based on PC analysis, we found that PC1 is most highly represented by the cancer cells within the liver. Then, upon projecting PC1 from MetMap onto our KPC dataset, we found that PC1 was recapitulated by the KPC samples from liver. This new observation supports that site-dependent differences in our RNAseq data is, at least in part, driven by signals specifically within the tumor cells and now has been added as a part of the Supplementary Figure 13 in the revised manuscript.

**MetMap PC projected onto KPC RNAseq data.** PC1, most highly represented in the liver metastatic cancer cells from MetMap data, is projected onto the KPC data using projectR and is well recapitulated in the KPC liver samples.

(3) Also, our extended CyTOF analysis added in the process of revision, as shown in Supplementary Figure 11, has validated the association of *Cxcl12* and *Ccl27* expression from the liver-specific KPC cells.
(4) Finally, in the process of our revision, we have also added the aforementioned IHC data, which further characterizes the metastatic immune microenvironment that are specific to intratumoral vs. adjacent normal regions. Together, these revisions help to delineate the cellular sources of the differences in the signals between the lung and liver metastases.
(5) As for FGL1, it is recently identified as a ligand for the checkpoint molecule LAG3, and as we have already cited in the manuscript, it has also been established in the literature to be secreted from the liver parenchyma (Wang et al., Cell 2019, PMID 30580966). This is also consistent with our data having shown high FGL1 expression in the liver samples from the RNAseq analysis of the non-immune compartment. This had been elaborated in the second paragraph of the discussion section in the original manuscript.
4. *There is no statistical section to the manuscript.*

A statistical section is now added into the revised manuscript within the methods. As the reviewer suggested, we have now created a dedicated “Bioinformatics and Statistical Analysis” section to provide better clarity as it comprises of all of the statistical methods used for all datasets.

5. *The authors also study the immune microenvironment of human PDAC from rapid autopsy specimens/database and TCGA datasets. The IHC shown in Fig S11 would benefit from a cytokeratin stain to demonstrate where the tumor cells are. The images are not of high enough resolution to permit interpretation of the H&Es or IHC stains. The sample size to make conclusions on the intratumoral density of immune infiltrates in distinct lesions is small and differences are modest especially in light of the heterogeneity portrayed in Fig S12.*

Higher resolution images including cytokeratin stains are now included as suggested (within the revised Supplementary Figures). We recognize that a small number of samples is a limitation of the study and already included it within the discussion section in the second to last paragraph. However, we had also noted within the second paragraph of the introduction in the original manuscript that profiling of multiple matched metastatic site samples from the same patient is quite challenging because there is rarely ever a need for simultaneous multisite biopsy for clinical decision-making. Given the limited feasibility, we submit that what we were able to observe in our sample set is valuable.

6. *Images in Fig S13B and S13C are the same except PD-1 in B and Lag3 in C. It would be easier to interpret if the images were not duplicated and Lag3 image was included as part of B. The resolution of the images is insufficient to allow for interpretation. Hi-mag insets would help too. It also appears that the differences in CD8 and subsequent Lag3 may represent focal accumulation of CD8 T cells rather than diffuse infiltration. Does this match with histology in the mouse model? Is Lag3 also increased in the normal parenchymal regions adjacent to tumor lesions?*

Images were rearranged according to this suggestion. Higher resolution images (both at 10X and 20X separately) are now also included within the Supplementary Figure 12. Comparing to our new IHC images from the mouse samples now shown as Supplementary Figure 6, we do observe that much of the immune infiltration occurs as tightly packed accumulations (shorter distances from one another). We now added this description into the results of the revised manuscript within the final paragraph of the *Immune profiling with mass cytometry reveals enhanced immune activation in lung metastases* subsection.

7. *For the RNAseq from autopsy database, many of the immune cell coefficients analyzed were undetectable in samples lending to few interpretable specimens. How was the quality of the RNA analyzed considered - for example, presence of CD45 transcripts of CK19 transcripts? Its also not clear how the authors conclude from this dataset that "these findings from human sample datasets largely corroborate higher T cell density in the lung TME and the immune-suppressed state within the liver TME".*

We appreciate the reviewer’s concerns about this dataset and recognize while it provides an additional resource, there are several limitations. We have determined that the inclusion of this analysis ultimately does not significantly add value to the overall study. We have thus removed this portion of the study.

***Reviewer #3:*** *General comments*

*The manuscript by Ho and colleagues describes a study using CyTOF and RNA-seq data to analyze lung and tumor microenvironments in metastatic pancreatic cancer. The authors first used a mouse model and CyTOF to characterize the infiltrating immune cells in lung and liver metastasis and then RNA-seq to study the non-immune compartment. The TME in the lung was composed of a higher level of immune cell infiltration and activation, whereas the non-immune compartment of the liver TME was relatively immune-suppressed compared to the lung. Human samples from an autopsies were analyzed using IHC and another published RNA-seq data was analyzed using deconvolution algorithm and some of the findings were corroborated. Finally, TCGA data was analyzed using transfer algorithm and deconvolution.*

1. *Albeit the study is of potential interest and might have translational implications, there are several concerns with the methodology as well as with the data analysis as described below. Specifically, the impact of the model on the TME needs to be analyzed in depth. It is necessary to provide the complete mutational profile of the used cell line (e.g. using exome-seq) since it is well known that the 1) mutational load/neoantigen load is associated with increased immune cell infiltration, and 2) a number of cancer driver genes are associated with the immune contexture (see PMID: 29562192). This information is of utmost importance for the interpretation of the results.*

The KPC model is commonly used in pancreatic cancer research and has been extensively characterized. We acknowledge the importance of presenting the mutational profile of the cell line being used. Per reviewer’s suggestion, we have performed WES and a mutational + neoantigen analysis of our KPC line, which are now added as the new Supplementary Figure 1. The description of these results are in the first paragraph of the results in the subsection *Establishing models of hepatic and pulmonary metastatic pancreatic ductal adenocarcinoma.*

2. *Another concern is the use of a deconvolution method MIXTURE which is not yet published and apparently validated using only simulated and benchmark data sets (according to the bioRxiv manuscript). There are several recently published deconvolution methods that enable robust analysis (see review PMID: 31515541). Hence, the authors should reanalyze the data with a published method of choice.*

While we recognize that MIXTURE is based on a preprint reference (<https://doi.org/10.1101/726562>), the study directly compares MIXTURE against CIBERSORT, a well-published deconvolution method (also described in the reviewer’s suggested review paper). In contrast to many other methods, MIXTURE and CIBERSORT are based on v-SVR and are less susceptible to overestimation by false cell-type identification overall. When compared side-by-side in the referenced bioRxiv manuscript, MIXTURE was less susceptible to overestimation related to noise. Furthermore, the referenced bioRxiv manuscript demonstrates the use of MIXTURE not only in simulated and benchmark datasets, but also real-world HNSCC and melanoma patient samples compared side-by-side with the results from CIBERSORT, together suggesting the robustness of MIXTURE. Based on reviewer’s suggestion, we did perform the same analysis shown in Figure 5 using CIBERSORT (shown below). The results mirror that of MIXTURE but with a tendency to show higher cell abundances of immune cells, all of which were expected based on what was described in the bioRxiv study. Given these factors, we believe that keeping the MIXTURE analysis results in the manuscript as originally submitted is justified and have not edited this portion of the manuscript.

*Finally, several specific comments need to be addressed.*

*Specific comments*

3. *Several papers analyzed RNA-seq data from the TCGA with respect to the immune cell infiltration (e.g. PMID: 31433971). Are the results of the current study (page 10) confirming previous findings?*

We appreciate the reviewer’s suggestion to relate our findings with prior reports. While we have not seen a direct comparison between lung and liver TCGA datasets using the deconvolved immune cell abundances as we have done here, we see that our results do confirm the previous findings from the study suggested by the reviewer, in which the overall leukocyte fraction was higher in the lungs than the livers and much greater lymphocyte-depleted immune subtypes within the liver samples. This is now added into the first paragraph of the revised discussion.

4. *The ErbB finding (page 7, line 20) should be either discussed in more detail or omitted.*

We agree with the reviewer’s suggestion to omit. This observation related to ErbB is now removed from the manuscript as we have recognized that the finding is beyond the main scope of this paper.

5. *The interplay between the immune and non-immune compartments of the TME is intriguing. The authors should consider using the information from a ligand-receptor database CellPhone.DB (PMID: 32103204) in order to provide additional evidence.*

To implement CellPhone.DB, the input dataset is that of scRNA-seq, which we have not performed. We agree with the reviewer that the analysis of ligand-receptor interactions within the TME compartments would be very interesting. We have now elaborated on the importance of this concept and the use of CyTOF as a limitation for this type of analysis within the second to last paragraph of the revised discussion section. In that same vein, we have also expanded the same paragraph on how the use of other single cell approaches would significantly add to the understanding of the TME.

6. *One innovative aspect is the use of the transfer learning approach. In the original publication mouse scRNA seq data from developing retina was used. The use of the method in a different domain (mouse-human immune cells) needs thorough discussion.*

We appreciate the reviewer’s positive comments about this approach. We have thus expanded our discussion related to this methodology as recommended in the fourth paragraph of the revised discussion section.

**Second round of review**

**Reviewer 1**

Point 1. I can agree with the authors' reply here, and leave it to the Editor's discretion as to whether functional experiments are required or not.

Point 2. The authors have addressed this point in more than sufficient depth.

Point 3. Also here, the authors did a good job on addressing my concern.

Some comments that could be addressed in another revision

- Why do the authors in some cases plot all datapoints with a median/mean, in some cases a box plot with whiskers, and bar graphs (with and without standard error).

- The array of boxplots in Figures 1 and 2 make for a very cluttered image. Is there some way to reduce or relocate some legends? Especially the significance symbols are hard to interpret at first sight. Does every plot need the X-axis legends?

**Reviewer 2**

The authors have made significant revisions to their manuscript which seeks to understand how the anatomic site influences cancer cell biology and its surrounding microenvironment. Their focus is on differences between liver and lung tumors. They identify many tissue-specific differences, but also show that these differences may influence the immune response to cancer. Their studies used Cytof, IHC, RNAseq. Overall, minor revisions are suggested to improve the readability of the work.

1. In general, given that this is an entirely descriptive work, the authors should refrain from concluding functional underpinnings from their studies. For example, the authors say on page 13 line 3 that the "liver exhibited a relatively greater pro-tumor immune microenvironment compared to lung." "Pro-tumor" implies function - the authors could alternatively state that a "pro-tumor" signature was more pronounced in the liver compared to lung. This is just one example. Additional comments are as follows:

2. Reference 2 should be updated.

3. When describing cytof analyses, the authors should consider refraining from calling this "liver TME" or "lung TME" as their analysis includes not only the TME but also the adjacent normal microenvironment. Alternatives might be to consider calling it the "composite tumor/liver microenvironment" or "metastatic liver microenvironment" for example.

4. On page 9 line 17-19, the authors state that the findings demonstrate that the non-immune compartment of the liver TME creates a "relatively immune-suppressed environment" compared to that of the lung. Demonstration of immune suppression and comparison functionally between the lung and liver is not assessed. The authors should consider rephrasing their conclusion based on the data.

5. On page 10, line 5-7: The authors comment on "abundance" with respect to data that is presented as "frequency". Abundance is not being measured.

6. In new data, the authors now show results for cytof analyses on liver and lung for two additional cell lines. The cell lines selected are 2838c3 and 6419 which have been described as T cell and DC infiltrated versus non-infiltrated , respectively, in prior work referenced by the authors (ref 25). This prior work involved subcutaneous tumors which differs from the studies performed by the authors. However, in the presented studies it appears that the authors find that anatomic location may be a fundamental determinant of this biology. For example, it appears that DC frequencies are low in 2838 when implanted into lung and high in the liver independent of the tumor line. This is also true for various T cell subsets presented. The authors should consider commenting on this observation in the discussion. Further. the text on page 10 line 5-7 appears incorrect and the direct opposite to what is shown in Figure 1. Is the legend backwards for lung and liver? Otherwise, the data in Suppl Fig 11 is opposite and comment should be made as B cells are increased here in the lung, and T cells and DC are increased in the liver.

**Reviewer 3**

The authors addressed the major issues I raised in the original review satisfactorily. Specifically, exome-seq of the KPC model was carried out, and additional analyses (CIBERSORT) were performed. Also the specific comments and suggestions were included and thereby considerably improved the manuscript.

**Authors Response**

**Point-by-point responses to the reviewers’ comments:**

Reviewer 1:

Point 1. I can agree with the authors' reply here, and leave it to the Editor's discretion as to whether functional experiments are required or not.

Point 2. The authors have addressed this point in more than sufficient depth.

Point 3. Also here, the authors did a good job on addressing my concern.

Some comments that could be addressed in another revision

- Why do the authors in some cases plot all datapoints with a median/mean, in some cases a box plot with whiskers, and bar graphs (with and without standard error).

- The array of boxplots in Figures 1 and 2 make for a very cluttered image. Is there some way to reduce or relocate some legends? Especially the significance symbols are hard to interpret at first sight. Does every plot need the X-axis legends?

*RESPONSE: Both of these comments pertain to data presentation within the figures. We appreciate the reviewer’s suggestion to improve this further. Regarding the reviewer’s first point, the main manuscript Figures 1-6 have been reviewed to assess where consistency can be improved. Specifically, while most figures use box plots, Figure 2B had utilized dot plots. Thus, it has now been replaced with box plots.*

*Furthermore, to address the reviewer’s second concern about Figures 1 and 2, the arrays of box plots from CyTOF analysis have been replaced with heatmaps and accompanying radar plots. We believe that this simplifies the demonstration of the data and thank the reviewer for the suggestion.*

Reviewer 2:

The authors have made significant revisions to their manuscript which seeks to understand how the anatomic site influences cancer cell biology and its surrounding microenvironment. Their focus is on differences between liver and lung tumors. They identify many tissue-specific differences, but also show that these differences may influence the immune response to cancer. Their studies used Cytof, IHC, RNAseq. Overall, minor revisions are suggested to improve the readability of the work.

1. In general, given that this is an entirely descriptive work, the authors should refrain from concluding functional underpinnings from their studies. For example, the authors say on page 13 line 3 that the "liver exhibited a relatively greater pro-tumor immune microenvironment compared to lung." "Pro-tumor" implies function - the authors could alternatively state that a "pro-tumor" signature was more pronounced in the liver compared to lung. This is just one example.

*RESPONSE: We appreciate this concern and have revised the manuscript to carefully improve the language when describing the results. For example, words like “demonstrate” noted at two places have been replaced with “imply” and “suggest”. We have also changed the specific sentence the reviewer pointed out to following: “Our analysis suggests that for a given tissue site, particular pathways are involved similarly in modulating – suppressing or stimulating – the immune response to different tumor types. In all of the datasets analyzed, liver exhibited a relatively greater presence of features that characterize an immune-suppressive pro-tumor immune microenvironment compared to lung.”*

Additional comments are as follows:

2. Reference 2 should be updated.

*RESPONSE: Reference 2 has been updated along with the referenced datapoint in the introduction.*

3. When describing cytof analyses, the authors should consider refraining from calling this "liver TME" or "lung TME" as their analysis includes not only the TME but also the adjacent normal microenvironment. Alternatives might be to consider calling it the "composite tumor/liver microenvironment" or "metastatic liver microenvironment" for example.

*RESPONSE: This is an important point. We agree and have revised the manuscript to replace any generalized use of the term “TME” with “metastatic microenvironment” or the deletion of that term when appropriate in the context of the results described.*

4. On page 9 line 17-19, the authors state that the findings demonstrate that the non-immune compartment of the liver TME creates a "relatively immune-suppressed environment" compared to that of the lung. Demonstration of immune suppression and comparison functionally between the lung and liver is not assessed. The authors should consider rephrasing their conclusion based on the data.

*RESPONSE: We agree with the potential of the current language to seemingly overstate our findings. As alluded to earlier, the word “demonstrate” has been revised to “suggest” to resolve this issue.*

5. On page 10, line 5-7: The authors comment on "abundance" with respect to data that is presented as "frequency". Abundance is not being measured.

*RESPONSE: We appreciate this comment, and we have replaced the word “abundance” with “proportion” (or similar derivative) to improve clarity in the description of our results.*

6. In new data, the authors now show results for cytof analyses on liver and lung for two additional cell lines. The cell lines selected are 2838c3 and 6419 which have been described as T cell and DC infiltrated versus non-infiltrated , respectively, in prior work referenced by the authors (ref 25). This prior work involved subcutaneous tumors which differs from the studies performed by the authors. However, in the presented studies it appears that the authors find that anatomic location may be a fundamental determinant of this biology. For example, it appears that DC frequencies are low in 2838 when implanted into lung and high in the liver independent of the tumor line. This is also true for various T cell subsets presented. The authors should consider commenting on this observation in the discussion. Further. the text on page 10 line 5-7 appears incorrect and the direct opposite to what is shown in Figure 1. Is the legend backwards for lung and liver? Otherwise, the data in Suppl Fig 11 is opposite and comment should be made as B cells are increased here in the lung, and T cells and DC are increased in the liver.

*RESPONSE: We agree with this observation and found it to be interesting. However, we believe that the comparison across the KPC cell line models in how they may contrast with the findings from the subcutaneous model in the referenced publication is beyond the scope of this study. Thus, we have not conducted studies to directly compare the subcutaneous and hemispleen liver/tail vein lung models in our hands for the different cell lines and would not be able to provide any data-driven discussion. Concerning the second comment, while the legends were not incorrect, we have recognized that the figures may be confusing to interpret as commented by another reviewer also. We have thus revised Figures 1 and 2 into heatmaps and accompanying radar plots. We believe that this change has improved the data presentation significantly.*

Reviewer 3:

The authors addressed the major issues I raised in the original review satisfactorily. Specifically, exome-seq of the KPC model was carried out, and additional analyses (CIBERSORT) were performed. Also the specific comments and suggestions were included and thereby considerably improved the manuscript.

*RESPONSE: We thank the reviewer for the time and feedback.*
